# Mitochondria-targeted nanoplatforms for enhanced photodynamic therapy against hypoxia tumor

**DOI:** 10.1186/s12951-021-01196-6

**Published:** 2021-12-20

**Authors:** Jiexin Wen, Yong Luo, Hui Gao, Liang Zhang, Xiang Wang, Ju Huang, Tingting Shang, Di Zhou, Dong Wang, Zhigang Wang, Pan Li, Zhaoxia Wang

**Affiliations:** 1 Department of Ultrasound, National Clinical Research Center for Child Health and Disorders, Ministry of Education Key Laboratory of Child Development and Disorders, Children’s Hospital of Chongqing Medical University, Chongqing, 400014 People’s Republic of China; 2 Department of Ultrasound, The First People’s Hospital of Chongqing Liang Jiang New Area, Chongqing, 401121 People’s Republic of China; 3 Chongqing Key Laboratory of Ultrasound Molecular Imaging, Institute of Ultrasound Imaging, The Second Affiliated Hospital, Chongqing Medical University, Chongqing, 400010 People’s Republic of China; 4 Department of Ultrasound, The Third Affiliated Hospital, Chongqing Medical University, Chongqing, 401120 People’s Republic of China; 5 Department of Radiology, The First Affiliated Hospital, Chongqing Medical University, Chongqing, 400042 People’s Republic of China; 6 Department of Ultrasound, The First Affiliated Hospital, Chongqing Medical University, Chongqing, 400042 People’s Republic of China

**Keywords:** Hypoxic tumor, 3-Bromopyruvate, Respiration inhibition, Photodynamic therapy, Nanomedicine

## Abstract

**Background:**

Photodynamic therapy (PDT) is a promising therapeutic modality that can convert oxygen into cytotoxic reactive oxygen species (ROS) via photosensitizers to halt tumor growth. However, hypoxia and the unsatisfactory accumulation of photosensitizers in tumors severely diminish the therapeutic effect of PDT. In this study, a multistage nanoplatform is demonstrated to overcome these limitations by encapsulating photosensitizer IR780 and oxygen regulator 3-bromopyruvate (3BP) in poly (lactic-co-glycolic acid) (PLGA) nanocarriers.

**Results:**

The as-synthesized nanoplatforms penetrated deeply into the interior region of tumors and preferentially remained in mitochondria due to the intrinsic characteristics of IR780. Meanwhile, 3BP could efficiently suppress oxygen consumption of tumor cells by inhibiting mitochondrial respiratory chain to further improve the generation of ROS. Furthermore, 3BP could abolish the excessive glycolytic capacity of tumor cells and lead to the collapse of ATP production, rendering tumor cells more susceptible to PDT. Successful tumor inhibition in animal models confirmed the therapeutic precision and efficiency. In addition, these nanoplatforms could act as fluorescence (FL) and photoacoustic (PA) imaging contrast agents, effectuating imaging-guided cancer treatment.

**Conclusions:**

This study provides an ideal strategy for cancer therapy by concurrent oxygen consumption reduction, oxygen-augmented PDT, energy supply reduction, mitochondria-targeted/deep-penetrated nanoplatforms and PA/FL dual-modal imaging guidance/monitoring. It is expected that such strategy will provide a promising alternative to maximize the performance of PDT in preclinical/clinical cancer treatment.

**Graphical Abstract:**

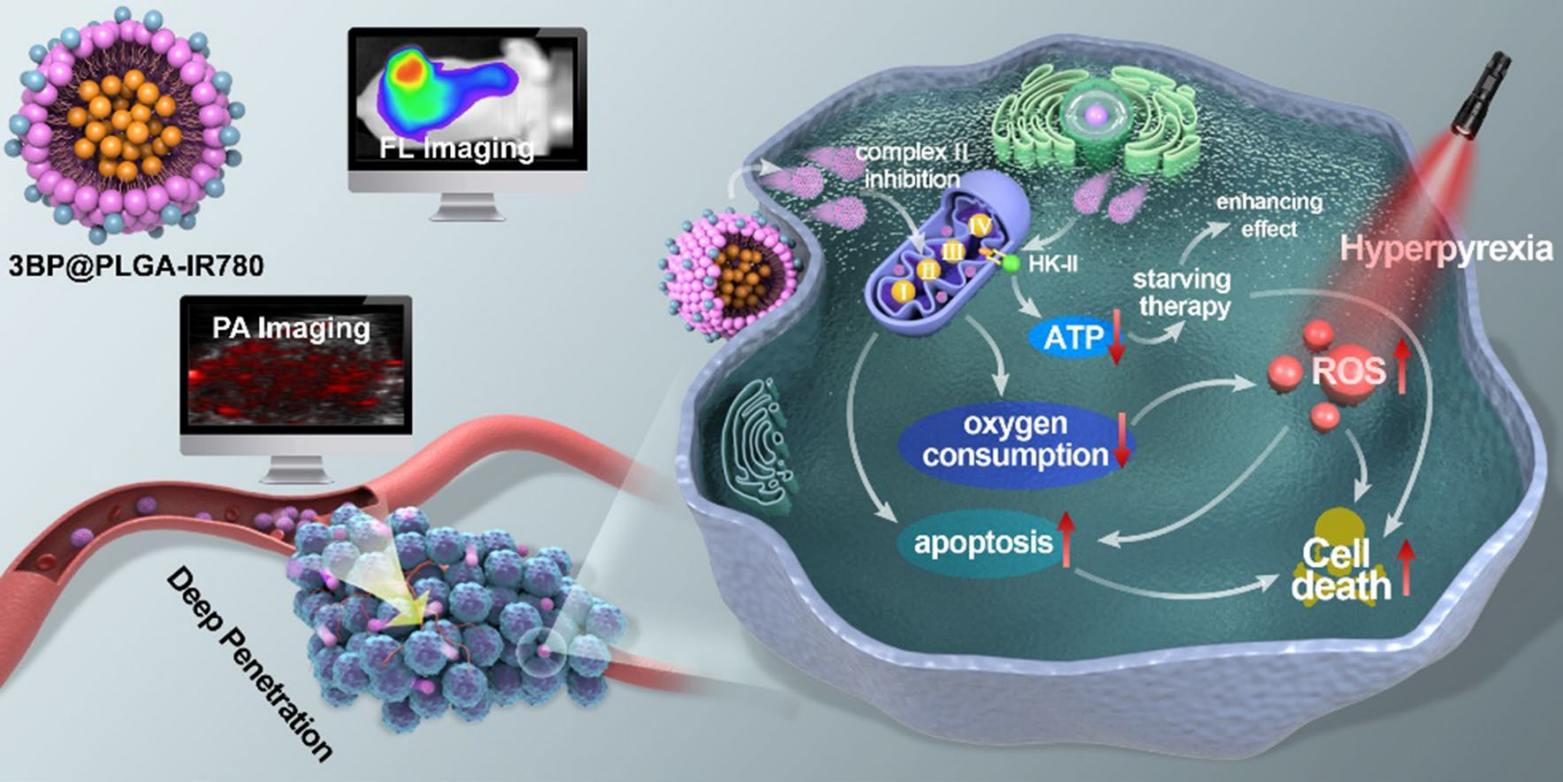

**Supplementary Information:**

The online version contains supplementary material available at 10.1186/s12951-021-01196-6.

## Introduction

Photodynamic therapy (PDT) is an evolving and promising therapeutic modality for its non-invasiveness, high selectivity, and low systemic toxicity compared to traditional chemotherapy and radiotherapy. Encouragingly, PDT has proven clinically to be an efficient therapeutic option for the treatments of esophageal cancer, lung cancer, and brain cancer, etc*.* [1]. PDT usually involves three elements including a tumor-localizing photosensitizer (PS), oxygen, and light irradiation at a certain wavelength [2, 3]. Upon excitation, the PS transmits the energy from light to oxygen molecules in tumor microenvironment, consequently producing reactive oxygen species (ROS) which kill tumor cells directly via inducing apoptosis and/or necrosis [4, 5].

The therapeutic effect of PDT largely depends on the amount of oxygen in the tumor. However, most solid tumors are in a state of hypoxia, namely an inadequate oxygen supply, which is mainly attributed to the deteriorating microenvironments, disturbed microcirculation, and aberrant tumor propagation [6, 7]. Clinical studies have proven that hypoxia is one of the main features that lead to the resistance to radiotherapy and chemotherapy [8, 9]. So as to PDT, it is well known that PDT requires the presence of oxygen [5, 7, 10]. To make matters worse, these hypoxic cells are reported to be more resistant to ROS than aerobic cells as well [11, 12]. To solve this problem, some strategies have been designed to alleviate hypoxia in tumor environment, many of which are developed to directly deliver oxygen molecules (O_2_) or hydrogen peroxide catalysts to tumor tissues with nanocarriers [10, 13, 14]. Although with preliminary successes, there remains insufficiencies, such as limited oxygen loading capacity, pre-mature oxygen release and low oxygen production effciency [15]. Bearing these in mind, aiming at the reduction of oxygen consumption is an insightful bypass to discourage tumor hypoxia.

Mitochondria-associated oxidative phosphorylation (OXPHOS) accounts for the predominant consumption of oxygen [16]. Interruption on this loop can potentially suppress O_2_ metabolism [17, 18]. By introducing 3-Bromopyruvate (3BP), a small-molecule pyruvate mimetic and anticancer alkylating agent that has also been reported to be a biocompatible antitumor agent [19], mitochondrial respiratory chain will be blocked [20, 21]. Such perturbation in the electron flow induces the decrease in oxygen consumption and restores the oxygen level that counters hypoxia [22]. In the meantime, hexokinase, catalyzing the essential first step of glycolysis is also an important target of 3BP [22, 23]. 3BP has pronounced inhibitory effects on multiple metabolic interactions, especially on the excessive glycolysis mediated by the over-expressed hexokinase type II (HK-II) in tumor cells, which will lead to the collapse of adenosine triphosphate (ATP) production [23, 24]. The starvation caused by 3BP will malnourish tumor cells and turned them into less defended therapeutic measures [25, 26]. Furthermore, HK-II is predominantly integrated with voltage dependent anion channel (VDAC) on the outer membrane of mitochondria [27, 28], which is of great significance to maintain mitochondrial membrane potential [29]. Thus 3BP-induced inhibition of HK-II can cause dissipation of mitochondrial membrane potential and induce the mitochondrial apoptotic pathway [30, 31]. The O_2_ consumption suppresion, stravation and enhanced mitochondria-associated apoptosis simutaneously render cancer cell more susceptable towards PDT. However, to properly design a carrier allowing on-target delivery remains a critical challenge.

**Scheme 1:**
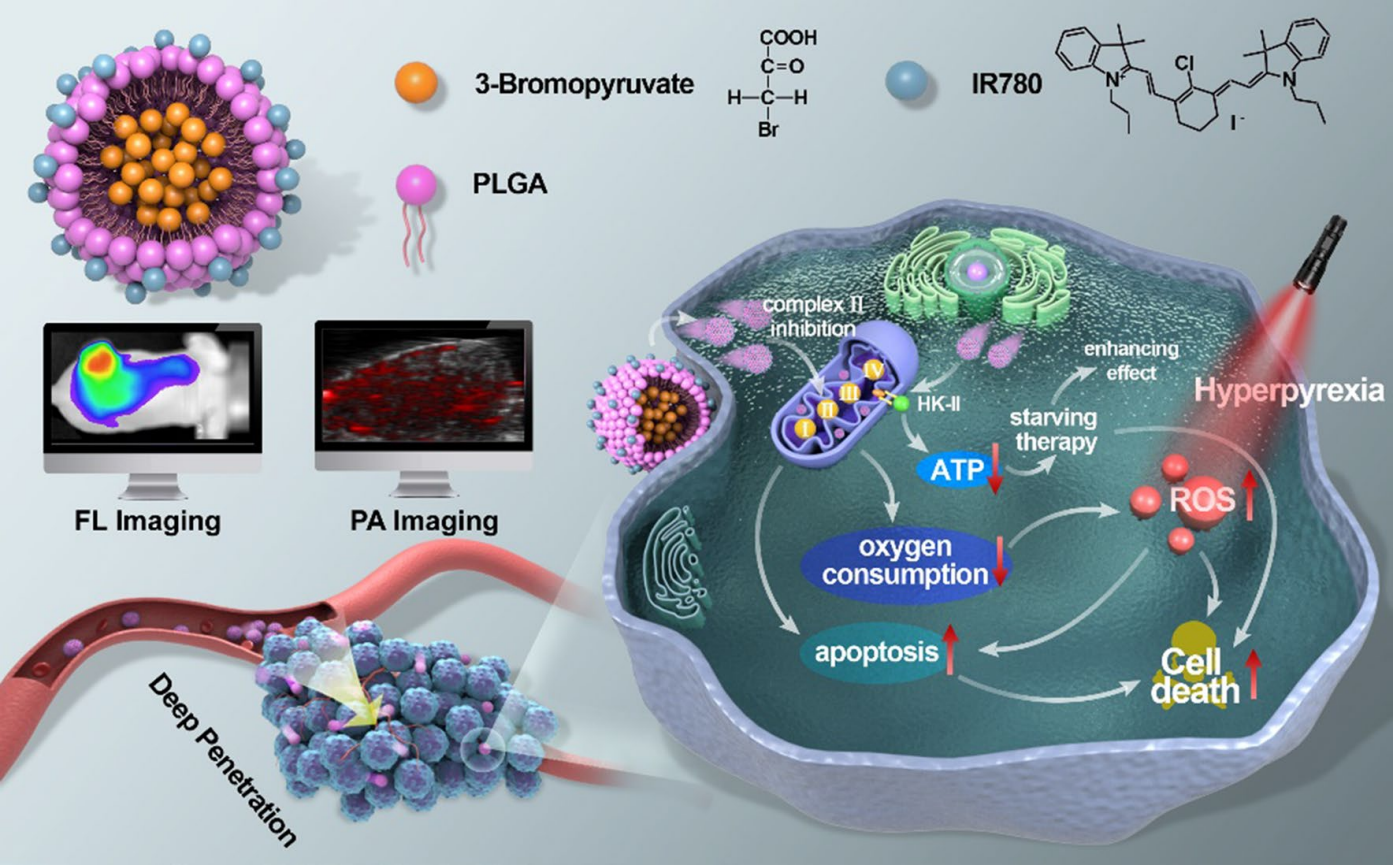
Schematic illustration of multifunctional 3BP@PLGA-IR780 nanoplatform, including concurrent oxygen consumption reduction, oxygen-augmented PDT, energy supply decrease, mitochondria-targeted/deep-penetrated capability and PA/FL dual-modal imaging guidance

To meet this need, in this study, we rationally contrustructed a core/shell structrued poly(lactic-co-glycolic acid) (PLGA) nanoplatform, with 3BP encapsulated in the core and IR780 loaded in the shell (designated as 3BP@PLGA-IR780), for enhanced PDT (Scheme 1). IR780, a lipophilic small molecular with characteristically considerable absorption and fluorescence in the near-infrared (NIR) wavelength region [32, 33], is used as a PS. These nanoplatforms could selectively accumulate and penetrate deeply in tumor tissues endowed by the nature of IR780 [34, 35]. Specially, 3BP@PLGA-IR780 could retain preferentially at intracellular mitochondria, presenting excellent locations for organelle-targeted PDT [10, 32, 36, 37]. Mitochondrion, a vital complex intracellular organelle, plays key roles in energy metabolism, production of ROS associated with oxidative stress and regulation of apoptosis [38, 39]. Thus, mitochondria have gained recognition as a viable subcellular target to enhance efficacy of anticancer treatments [10, 36]. To optimize therapeutic precision, we took advantage of the fluorescence (FL) and photoacoustic (PA) imaging capability of IR780 to monitor the tumor accumulation of these nanoplatforms [33, 40]. Thus, highly efficient antitumor therapy can be achieved by concurrent oxygen consumption reduction, oxygen-augmented PDT, energy supply decrease, mitochondria-targeted/deep-penetrated nanoplatforms and PA/FL dual-modal imaging guidance. In practice, systematic in vitro and in vivo evaluations have been conducted in this work to demonstrate the efficacy of 3BP@PLGA-IR780 for amplified PDT.

## Materials and methods

### Materials and reagents

IR780 iodide, 3BP, and (2′,7′-dichlorofluorescin diacetate (DCFH-DA) were purchased from Sigma-Aldrich (St. Louis, MO, USA). PLGA-PEG-3.4 k was purchased from Xi’an Ruixi Biological Technology Co., Ltd. (Xi’an, China). Cell-Counting Kit-8 (CCK-8), calcein AM, and pyridine iodide (PI) were obtained from Daigang Biological Engineering Co., Ltd. (Jinan, China). Dichloromethane (CHCl_2_) was purchased from Chuandong Chemical Co. Ltd. (Chongqing, China). Nuclear dye 4,6-Diamidino-2-phenylindole (DAPI), enhanced ATP assay kit and 1,1'-dioctadecyl-3,3,3′,3′-tetramethylindocarbocyanine perchlorate (Dil) were purchased from Beyotime Biotechnology (Shanghai, China). Singlet Oxygen Sensor Green (SOSG) and MitoTracker^®^ Deep Red FM were purchased from Thermo Fisher Scientific (Invitrogen). Dimethyl sulfoxide (DMSO) was purchased from Shanghai Aladdin Biochemical Technology Co., Ltd. (Shanghai, China). All other reagents were of or above analytical grade and used as received without further purification.

### Synthesis of 3BP@PLGA-IR780

3BP@PLGA-IR780 were synthesized via a water–oil-water double emulsion protocol. Briefly, 50 mg of PLGA and 1 mg of IR780 were dissolved in 2 mL of CHCl_2_, and 3BP (50 mg) dissolved in phosphate buffer saline (PBS) (200 μL) was added to the above solution. The polymeric solution was emulsified with an ultrasonic processor (Sonics & Materials Inc., Danbury, CT, USA) operating at a power intensity of 100 W for 3 min (on 5 s, off 5 s). Then, 9 mL of polyvinyl acetate (PVA) solution was added into the formed emulsion. Then, the emulsion was further subjected to a probe sonication (50 W, on 5 s, off 5 s). Finally, 10 mL of isopropyl alcohol was added to the above solution, and the mixture was stirred mechanically to evaporate the CHCl_2_ completely. The fabricated 3BP@PLGA-IR780 nanoplatforms were collected and stored at 4 °C for further use. The same procedure was made to other nanoplatforms, but differentiating the drug.

### Characterizations of 3BP@PLGA-IR780

The morphologies of 3BP@PLGA-IR780 and 3BP@PLGA were observed by TEM (Hitachi H-7600, Japan) and SEM (JEOL JSM-7800F, Japan). A laser particle size analyzer system (Nano, ZS90, Malvern instrument Ltd.) was used to determine the size distribution and surface zeta potential of 3BP@PLGA-IR780 and 3BP@PLGA. UV–vis–NIR absorption spectra of IR780 (0.125, 0.25, 0.5, 1.0, 2.0, 3.0, 4.0, and 5.0 μg mL^−1^), 3BP (0.1, 0.2, 0.4, 0.6, 0.8 and 1.0 mg mL^−1^), 3BP@PLGA-IR780 and 3BP@PLGA were recorded using the SpectraMax Paradigm Multi-Mode Microplate Reader (SpectraMax M3, Molecular Devices, USA). The Drug Loaded Efficiency was then calculated by the following equation.

$${\text{Drug\, Loaded\, Efficiency}} = \left( {{\text{Initial \,Weight \,of \,Drug}}{-}{\text{unloaded \,Drug}}} \right)/{\text{Initial \,weight \,of \,Drug}}$$

In vitro photodynamic performance of 3BP@PLGA-IR780 was evaluated by a typical SOSG assay. 3BP@PLGA-IR780 dispersed in PBS containing SOSG (25 μM) at different IR780 concentrations (2, 4, 6, 8 and 10 μg mL^−1^) was added into a cuvette, and exposed to an 808 nm laser irradiation (1.0 W cm^−2^) for different time durations (0, 10, 30, 60, 120 and 180 s). The fluorescence intensity curves were recorded by using the SpectraMax Paradigm Multi-Mode Microplate Reader (λexcitation = 488 nm).

The photo-stability of 3BP@PLGA-IR780 was evaluated next. Free IR780 (pre-dissolved in a small amount of DMSO) and 3BP@PLGA-IR780 in PBS were irradiated by an 808 nm laser (1.0 W cm^−2^) for 10 s each time. The absorbance of the samples at 789 nm were recorded with a microplate reader at 0, 10, 20, 30, 40, 50 and 60 s after the irradiation. The absorbance values were then normalized as A/A_0_ (A is the absorbance of the sample and A_0_ is the absorbance of the sample before irradiation). Free IR780 and 3BP@PLGA-IR780 in PBS were placed in a completely dark environment, and the absorbance of the samples at 789 nm were recorded on day 0, 1, 3, and 4. The absorbance values were also normalized.

### Cell culture and 4T1 tumor-bearing mice model

Murine breast cancer line 4T1 cells were obtained from Shanghai Zhong Qiao Xin Zhou Biotechnology Co., Ltd. All-female Balb/c nude mice (4–6 weeks) were purchased from Chongqing Medical University. All the experiments and procedures were performed under guidelines approved by the Institutional Animal Care and Use Committee of Chongqing Medical University. To establish 4T1 tumor-bearing mice models, 1 × 10^6^ 4T1 cells were suspended in 100 µL serum-free medium and then injected subcutaneously to the flanks of the nude mice.

### Intracellular uptake and mitochondria location of 3BP@PLGA-IR780

The intracellular uptake of 3BP@PLGA-IR780 was detected by confocal laser scanning microscope (CLSM) (Nikon A1, Japan) and flow cytometry (CytoFLEX, USA). Briefly, 4T1 cells were seeded in a CLSM-specific dish at a density of 1 × 10^5^ cells per well. After 24 h incubation, the culture medium was replaced with a serum-free medium containing red Dil (λexcitation/λemission = 549 nm/565 nm) labeled 3BP@PLGA or 3BP@PLGA-IR780 (PLGA equivalence: 200 μg mL^−1^). After different intervals of incubation (0.5 h, 1 h, 2 h, and 4 h), the cells were washed with PBS for three times to remove those nanoplatforms which had not yet entered tumor cells, fixed with 4% polyformaldehyde, and stained with blue DAPI (λexcitation/λemission = 340 nm/488 nm) for 10 min. Then, the cells were observed by CLSM. Additionally, the quantitative intracellular uptake (red fluorescence intensity) was further analyzed with flow cytometry. After 4 h incubation with Dil-labeled 3BP@PLGA-IR780 or 3BP@PLGA, to investigate the mitochondrial localization of these nanoplatforms, mitochondria of 4T1 cells were sequentially stained with MitoTracker^®^Deep Red FM (λexcitation/λemission = 644 nm/665 nm, the color was set as red during CLSM observation) for another 30 min. Then the cells were rinsed with PBS and observed with CLSM. In addition, intracellular uptake of 3BP@PLGA-IR780 by MCF-7 and MBA-MD-231 cells after different intervals of incubation (1 h and 4 h) was further evaluated by CLSM observation.

### Mitochondrial membrane potential and ATP measurement

Mitochondrial membrane potential was analyzed with JC-1 staining. When the mitochondrial membrane potential is high, representing healthy cells, JC-1 aggregates in the matrix of the mitochondria to form J-aggregates, which produces red fluorescence; when the mitochondrial membrane potential is low, representing apoptotic cells, JC-1 cannot aggregate in the matrix and the monomer remains at this time, which produces green fluorescence. 4T1 cells were cultured in confocal cell-culture dishes as mentioned in 2.5. 3BPA@PLGA-IR780 and 3BP@PLGA were added in serum-free culture media (PLGA equivalence: 200 μg mL^−1^), and the cells were further incubated for another 4 h. The mitochondria were then stained with JC-1 for 20 min. Cells were washed twice with PBS prior to CLSM imaging. Cellular ATP levels were further detected with an Enhanced ATP Assay Kit performed according to the standard protocol.

### Dissolved oxygen measurement

4T1 cells were seeded in 12-well plates (1.2 × 10^5^/well) and cultured overnight. These cells were treated with 3BPA@PLGA-IR780 and 3BP@PLGA (PLGA equivalence: 200 μg mL^−1^, n = 3), and 1 mL of liquid paraffin oil was added to each well. After incubating for 4 h in a 37 °C incubator, the oxygen content in the culture medium was measured with a dissolved oxygen meter (Mettler Toledo, Switzerland).

### ROS generation and in vitro cytotoxicity of 3BP@PLGA-IR780

For the detection of intracellular ROS, pre-cultured 4T1 cells were randomly divided into six groups (control, Laser only, 3BP@PLGA, 3BP@PLGA-IR780, PLGA-IR780 + Laser and 3BP@PLGA-IR780 + Laser). The corresponding cells were treated with 3BPA@PLGA-IR780 or 3BP@PLGA (PLGA equivalence: 200 μg mL^−1^) and further incubated for 4 h. Subsequently, these cells were incubated with ROS probe DCFH-DA (30 μM for CLSM detection, 10 μM for flow cytometry analysis) for 10 min, followed by laser irradiation (1.0 W cm^−2^, 5 min) in ice bath (excluding the photothermal effect). Then CLSM observation were conducted and the fluorescence intensities of each group were measured by flow cytometry.

To test the therapeutic effects of 3BP@PLGA-IR780, 4T1 cells were seeded in a 96-well plate (1 × 10^4^ cells per well) for 24 h. Then, these 4T1 cells were subjected to following treatments: (1) control (saline), (2) Laser only (1.0 W cm^−2^, 5 min), (3) 3BP@PLGA, (4) 3BP@PLGA-IR780 only, (5) PLGA-IR780 + Laser, and (6) 3BP@PLGA-IR780 + Laser. All PDT treatments were conducted in ice-bath to cool down and keep the temperature under 42 °C to exclude the photothermal effect. Finally, the cell viabilities were evaluated using a standard CCK-8 kit. Besides, flow cytometry was also employed to analyze cell apoptosis induced by different treatments. To discriminate live and dead cells, the treated cells were costained with calcein-AM (2 μmol mL^−1^, λexcitation/λemission = 490 nm/515 nm) and PI (4.5 μmol mL^−1^, λexcitation/λemission = 530 nm/580 nm) for CLSM observation. The red fluorescence represents dead cells, while green fluorescence means living cells.

### In vivo biodistribution (FL imaging) and pharmacokinetics study of 3BP@PLGA-IR780

For the detection of in vivo biodistribution of 3BP@PLGA-IR780, mice bearing 4T1 tumors (n = 3) were intravenously injected with 3BPA@PLGA-IR780 saline solution (200 µL, the corresponding PLGA concentration was 10 mg mL^−1^). The in vivo FL imaging at varied time points was performed with a Living Imaging System and the average fluorescence intensities of tumors were measured by the system. Mice were sacrificed and the tumor masses, livers and spleens were collected 48 h after injection. The above tissues were sliced and stained with DAPI for fluorescence microscopy observation.

To study the pharmacokinetics of 3BP@PLGA-IR780, healthy mice and tumor-bearing mice were intravenously administrated with 3BP@PLGA-IR780 (PLGA equivalence: 10 mg mL^−1^, 200 μL). At different time points (0, 0.1, 1, 4, 12, 24, 48 and 72 h, n = 3) after the intravenous administration, blood was collected from the retro-orbital plexus, and the serum was obtained by centrifugation at 3000×*g* for 10 min. The amount of IR780 in serum was detected by a microplate reader. The pharmacokinetic parameters were calculated according to the dual-compartment pharmacokinetic model. In addition, the ex vivo fluorescence imaging of isolated tumors was performed at 1, 3, 5, 7 and 10 days (n = 3 at each time point) after intravenous injection of 3BP@PLGA-IR780.

### Deep penetration of 3BP@PLGA-IR780

The deep penetration capability of 3BP@PLGA-IR780 was evaluated in 3D tumor spheroid models. To establish the 3D tumor models, 4T1 cells (5 × 10^4^) were seeded in spheroid microplates and cultured for 7 days. The established 3D spheroids were treated with Dil-labeled 3BP@PLGA-IR780 or 3BP@PLGA (PLGA equivalence: 500 μg mL^−1^). After culturing for 12 h, the tumor spheroids were rinsed twice with PBS and then observed by CLSM.

For the evaluation of in vivo penetration, 4T1 tumor-bearing mice were treated with Dil-labeled 3BP@PLGA-IR780 or 3BP@PLGA (PLGA equivalence: 10 mg mL^−1^, 200 μL), and the tumors were harvested at 24 h post-injection. The tumor masses were sliced and an anti-CD31 antibody was used to stain blood vessels for fluorescence microscopy observation.

### PA imaging of 3BP@PLGA-IR780

To investigate the performance of 3BP@PLGA-IR780 as a PA contrast agent, both in vitro and in vivo experiments were conducted. Firstly, 3BP@PLGA-IR780 suspension was stimulated by a PA laser with excitation wavelength ranging from 680 to 970 nm. Then, PA values of different concentrations of 3BP@PLGA-IR780 (the corresponding IR780 concentrations were 10, 20, 30, 40, 50 µg mL^−1^) were measured, and corresponding PA images were attained. To evaluate the in vivo PA imaging performance, 4T1 tumor-bearing mice were intravenously injected with 200 μL of 3BP@PLGA-IR780 (PLGA equivalence: 10 mg mL^−1^). The tumor PA images were collected at different time points (0, 1, 2, 4, 6, 24, and 48 h, n = 3) and the corresponding PA intensities were analyzed by a Vevo LAZR System (Visual Sonics Inc., Canada).

### Detection of tumor hypoxia status

To detect the hypoxia status of the 4T1 tumor, tumor-bearing mice were randomly divided into three groups (n = 3) and injected with saline, 3BP@PLGA, and 3BP@PLGA-IR780 (PLGA equivalence: 10 mg mL^−1^, 200 μL), respectively. At 24 h post-injection, the tumor masses were collected, sliced and stained by the hypoxia-inducible factor (HIF-1α) antibody for the detection of HIF-1α expression. Meanwhile, the oxygenated hemoglobin level of tumors of each group was monitored by a Vevo LAZR PA Imaging System in oxy-hem mode. The average oxyhemoglobin saturation within tumors was analyzed by testing the ratios of oxygenated hemoglobin and deoxygenated hemoglobin. Furthermore, western blot analysis was performed to assess the expression of HIF-1α in tumors.

### In vivo synergistic tumor therapy

To evaluate the in vivo PDT efficacy of 3BP@PLGA-IR780, thirty 4T1 tumor-bearing mice were randomly separated into six groups (n = 5) as follows: (1) Control (intravenous injection of saline), (2) Laser only (1.0 W cm^−2^, 5 min), (3) 3BP@PLGA (intravenous injection of 3BP@PLGA), (4) 3BP@PLGA-IR780 (intravenous injection of 3BP@PLGA-IR780), (5) PLGA-IR780 + Laser (intravenous injection of PLGA-IR780 + laser exposure), and (6) 3BP@PLGA-IR780 + Laser (intravenous injection of 3BP-PLGA-IR780 + laser exposure). The corresponding PLGA concentrations were 10 mg mL^−1^ and the injection volume was 200 μL.

To exclude the photothermal effect, the laser irradiation was implemented for 40 s and subsequently followed by intervals to cool down to keep the tumor temperature under 42 °C. The above irradiation was repeated for 15 cycles. Mice treated with saline were regarded as the blank control group. Mice treated with laser only were used to show that laser irradiation without photosensitizers will not produce a therapeutic effect. Mice treated with 3BP@PLGA or 3BP@PLGA-IR780 only were used to demonstrate the effects of 3BP@PLGA or 3BP@PLGA-IR780 on tumors in vivo. Mice treated with PLGA-IR780 + Laser were regarded as the PDT group. Mice treated with 3BP@PLGA-IR780 + Laser were regarded as oxygen-augmented PDT group. The tumor volumes and bodyweight of mice were monitored every other day. At the end of treatments, the tumor tissues were dissected, weighed, and photographed. The main organs (heart, liver, spleen, lung, and kidney) and the tumor tissues were harvested and fixed in a 4% paraformaldehyde solution for histopathological analysis including Hematoxylin–eosin (H&E), TdT-mediated dUTP Nick-End Labeling (TUNEL), and proliferating cell nuclear antigen (PCNA) staining.

### Biosafety assessment

The biosafety of the 3BP@PLGA-IR780 was implemented on healthy *Kunming* mice. The mice were intravenously injected with 3BP@PLGA-IR780 (PLGA equivalence: 10 mg mL^−1^, 200 μL). The blood of the mice was collected at various time points (7, 15, and 30 days) for blood cell analysis and biochemical assays. The untreated mice were used as controls. Meanwhile, the major organs of the mice were collected and stained with H&E.

### Statistical analysis

All statistical analyses were performed with SPSS 20.0 software. Data were presented as mean ± standard deviation. The significance of the data is analyzed according to a Student’s t-test: *P < 0.05, **P < 0.01, ***P < 0.001.

## Results

### Synthesis and characterization of 3BP@PLGA-IR780

**Fig. 1:**
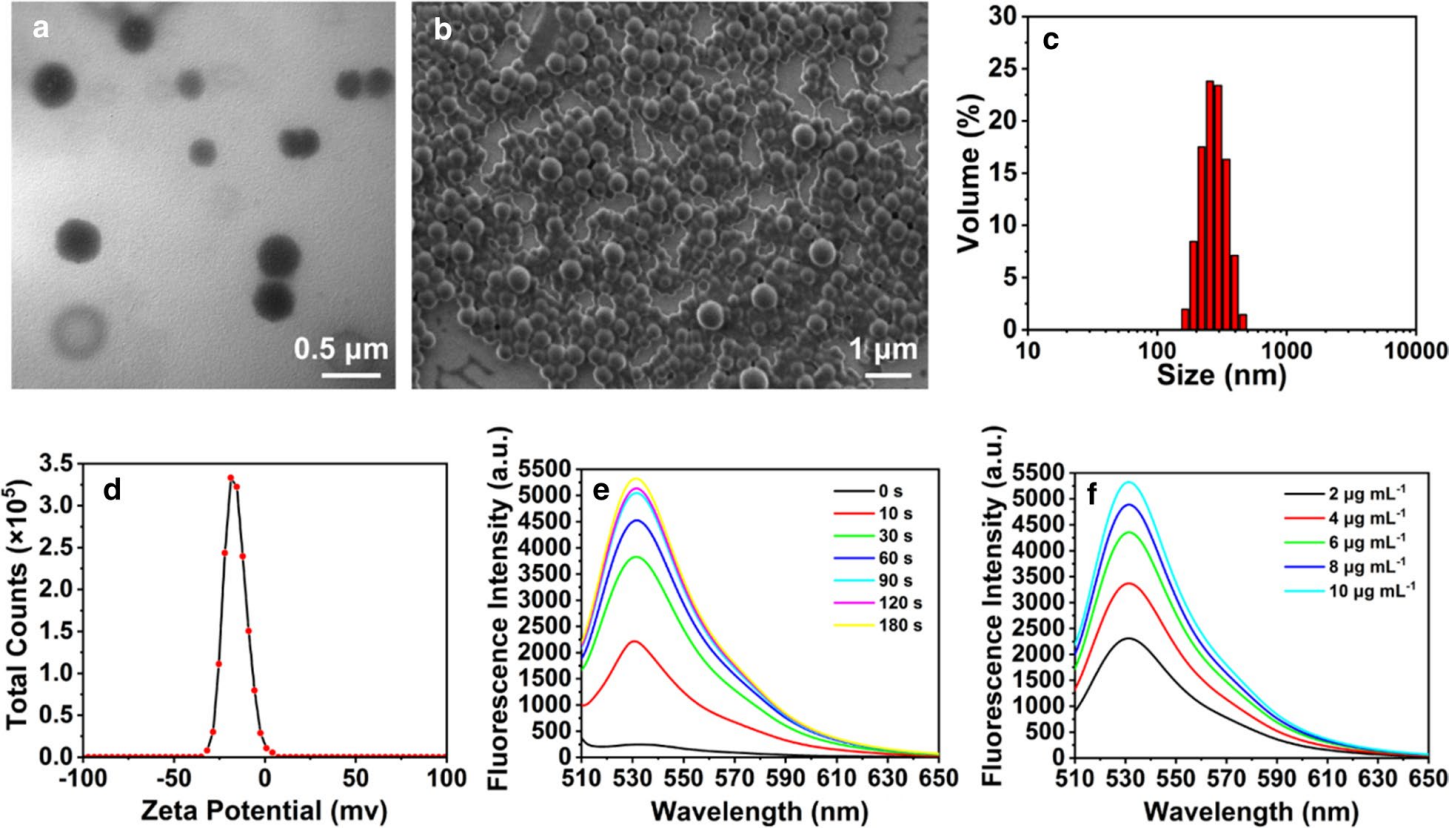
Characterizations of 3BP@PLGA-IR780. **a** SEM image, **b** TEM image, **c** hydrodynamic diameter, and **d** surface zeta potential of 3BP@PLGA-IR780. **e** Time-dependent ROS generation of 3BP@PLGA-IR780 detected by SOSG at a fixed IR780 concentration of 10 μg mL^−1^. **f** Concentration-dependent ROS generation of 3BP@PLGA-IR780 detected by SOSG

3BP@PLGA-IR780 was prepared via a double-emulsion in absence of light, with hydrophilic 3BP encapsulated inside the core and lipophilic IR780 in the lipid bilayer. Only 3BP was added in synthesis of 3BP@PLGA. Typical scanning electron microscopic (SEM) and transmission electron microscopic (TEM) images showed that the as-prepared 3BP@PLGA-IR780 and 3BP@PLGA nanoplatforms presented a spherical structure (Fig. 1a, b, Additional file 1: Fig. S1a and S1b). Measured by dynamic light scattering (DLS), the average hydrodynamic diameter of 3BP@PLGA-IR780 and 3BP@PLGA nanoplatforms were 252.8 ± 61.76 nm and 237.7 ± 51.08 nm, respectively (Fig. 1c and Additional file 1: Fig. S1c). And the zeta potential of 3BP@PLGA-IR780 and 3BP@PLGA nanoplatforms were − 16.2 ± 6.08 mV and − 19.6 ± 4.57 mV, respectively, which could be desirable for in vivo application (Fig. 1d and Additional file 1: Fig. S1d) [41]. The zeta potential of 3BP@PLGA-IR780 nanoplatforms was slightly higher than that of 3BP@PLGA, which may be related to the weak positive charge of IR780 loaded on the nanoplatforms. The UV–vis–NIR spectra of IR780 and 3BP exhibited a concentration-dependent manner, with obvious characteristic bands at 789 nm and 326 nm (Additional file 1: Fig. S2 and S3). As shown in Additional file 1: Fig. S4, PLGA loaded with IR780 showed an obvious absorption peak in accordance with the spectrum of pristine IR780 (Additional file 1: Fig. S2a), and 3BP-based PLGA including 3BP@PLGA-IR780 and 3BP@PLGA featured the similar peak of 3BP (Additional file 1: Fig. S3a), which demonstrated the successful loading of both IR780 and 3BP. The loading efficiencies of IR780 and 3BP in 3BP@PLGA-IR780 were calculated to be 86.86 ± 5.22% and 12.60 ± 2.58%, respectively.

IR780 was dissolved in CHCl_2_ during the synthesis of 3BP@PLGA-IR780 and embedded in PLGA shell, which could protect IR780 from spontaneous quenching. Considering that 3BP does not possess fluorescent property and its bioactivity would not be noticeably attenuated due to light quenching. We mainly focus on evaluating the photo-stability of IR780. It was evaluated by the changes in absorbance values of free IR780 and 3BP@PLGA-IR780 after laser exposure (Additional file 1: Fig. S5a). As shown in Additional file 1: Fig. S5b, IR780 in 3BP@PLGA-IR780 nanoplatforms was consumed more slowly than free IR780. After 60 s of laser irradiation, 3BP@PLGA-IR780 still maintained about 73.84% of the initial absorbance. In contrast, the free IR780 was severely photobleached (18.36%). It was also found that the absorbance intensity of 3BP@PLGA-IR780 did not change significantly after placing in the dark for 4 days, while the free IR780 almost lost its absorbance (Additional file 1: Fig. S5c and S5d). These results showed that after loading IR780 into 3BP@PLGA-IR780, its photo-stability can be significantly improved, which is of great significance for subsequent biological applications.

Since the prepared nanoplatforms were designed for boosting PDT efficiency, which was closely correlated with ROS generation. Therefore, in vitro ROS generation of 3BP@PLGA-IR780 was demonstrated by a singlet oxygen sensor SOSG fluorescence probe. At a fixed IR780 concentration of 10 μg mL^−1^, the fluorescence intensities increased with prolonged laser irradiation duration, indicating the excellent ROS generation of 3BP@PLGA-IR780 (Fig. 1e). Moreover, the fluorescence intensity changes also demonstrated a similar trend at various concentrations of 3BP@PLGA-IR780 within the same irradiation duration (Fig. 1f). The changes in SOSG fluorescence intensities indicated the potential of 3BP@PLGA-IR780 as a dependable PS to mediate PDT against cancer.

### Intracellular uptake of 3BP@PLGA-IR780

**Fig. 2:**
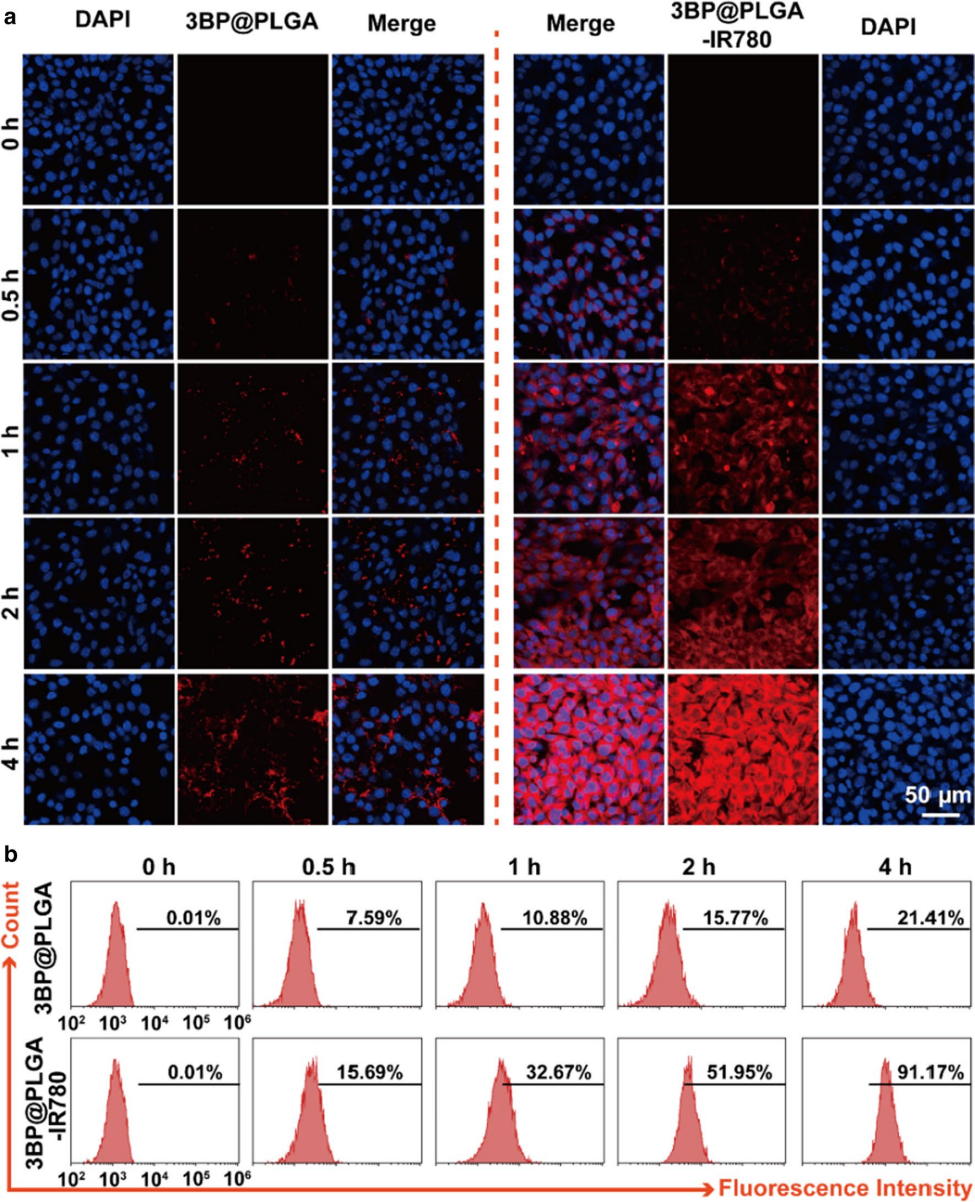
**a** CLSM images of intracellular uptake of 3BP@PLGA-IR780 (the red represents nanoplatforms and the blue represents cell nucleus). **b** Flow cytometry analysis of phagocytosis efficacy of 3BP@PLGA or 3BP@PLGA-IR780

Considering that these nanoplatforms may confront tremendous challenges including cell phagocytosis, which is detrimental to the subsequent therapeutic efficacy, in this work, we investigated the intracellular uptake behavior of 3BP@PLGA-IR780. 4T1 cells were incubated with Dil-stained 3BP@PLGA-IR780 or 3BP@PLGA suspensions and subsequently observed by CLSM. Tumor cells would uptake increasing nanoplatforms with prolonged incubation time, exhibiting growing fluorescence intensities. This tendency could be observed in both 3BP@PLGA group and 3BP@PLGA-IR780 group, as shown in Fig. 2a. However, more intensive red fluorescence of 3BP@PLGA-IR780 was observed in 4T1 cells and increased significantly over incubation time, while relatively weaker red fluorescence was detected in the 3BP@PLGA-treated group without IR780. Similarly, the flow cytometry analysis was consistent with CLSM images that the red fluorescence intensities in cells treated with 3BP@PLGA-IR780 were much stronger than that in the 3BP@PLGA group (Fig. 2b). In addition, intracellular uptake of 3BP@PLGA-IR780 by MCF-7 and MBA-MD-231 cells were further evaluated. Likewise, as shown in Additional file 1: Fig. S6, after 1 h and 4 h of incubation, red fluorescence of the 3BP@PLGA-IR780-treated groups was much higher than that of 3BP@PLGA without IR780. Those results suggested that IR780 played a key role in enhancing the intracellular uptake of the nano-systems and facilitating the accumulation of drugs in tumor tissue.

### Mitochondria-targeting capability of 3BP@PLGA-IR780 and synergetic effects of 3BP

**Fig. 3:**
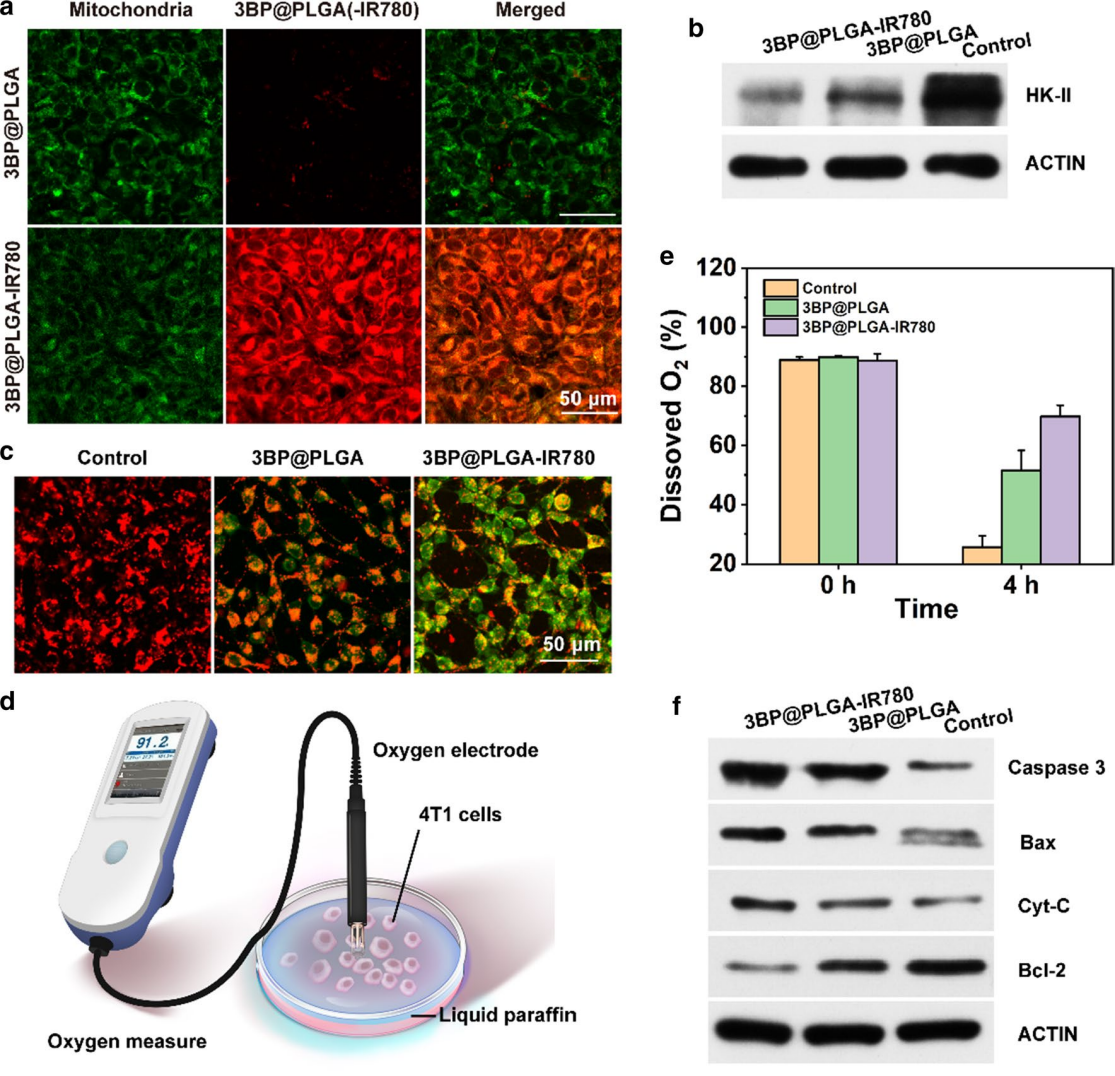
**a** CLSM images of 3BP@PLGA-IR780 colocalization with mitochondria (the red represents nanoplatforms and the green represents mitochondria. **b** Western blot results of HK-II expression in 4T1 cells after various treatments. **c** CLSM images of JC-1 stained-4T1 cells after co-incubating with 3BP@PLGA and 3BPA@PLGA-IR780 (the red indicates normal membrane potential, the green indicates declined membrane potential). **d** Schematic illustration of the measurement of dissolved O_2_ with an oxygen electrode. **e** The changes of dissolved O_2_ of cell culture medium after incubation with 3BP@PLGA or 3BP@PLGA-IR780 for 4 h. **f** The changes of apoptotic proteins in 4T1 cells after various treatments

Aiming at mitochondria, 3BP is a highly effective anti-tumor drug, but the clinical application of it is limited to some degree due to insufficient delivery and subsequent side-effects [19]. These multi-functional 3BP@PLGA-IR780 nanoplatforms offer an intriguing approach to tackle this dilemma. IR780 has the unique ability to target tumor mitochondria, not only enriches IR780-based nanoplatforms in the targeted region, but also enhances the focus of 3BP on mitochondria. The difference of subcellular localization of 3BP@PLGA-IR780 and 3BP@PLGA can be seen from Fig. 3a. As expected, 3BP@PLGA-IR780 were mainly internalized and retained in mitochondria after 4 h of incubation. In contrast, relatively poor accumulation of 3BP@PLGA in mitochondrial regions was observed (Fig. 3a), implicating the indispensable contribution of IR780 in the mitochondrial-targeting behavior. Mitochondrion is a vital cell organelle, and any damage or interruption on it can be lethal to cells. The mitochondria-targeting ability of 3BP@PLGA-IR780 may represent a promising approach in this regard [10, 29, 36, 42].

Effects of 3BP on mitochondria mainly include the inhibition of HK-II and the interference with the mitochondrial respiratory chain to cause mitochondrial dysfunction, which further leads to over-increased membrane permeability and induces apoptosis following mitochondrial pathways [22, 30, 31]. HK-II is an important target of 3BP [22]. Predominantly integrated with VDAC on the outer membrane of mitochondria [27, 28], HK-II is of great significance to maintain mitochondrial membrane potential and develop the Warburg phenotype in most types of cancer [27, 29]. 3BP can inhibit HK-II activity directly, which may due to the alkylating property of 3BP [30, 43]. In addition, 3BP can also reduce the expression of HK-II. The reduction in HK-II expression may result from a shortage of energy supply caused by metabolic perturbation [44]. The Western blot test results showed that the expression of HK-II decreased significantly after co-incubation with 3BP@PLGA or 3BP@PLGA-IR780, and the addition of IR780 made the expression of HK-II drop more significantly (Fig. 3b). The blocking effect to HK-II can further cause the separation of HK-II and VDAC, resulting in the shedding of HK-II from the outer mitochondrial membrane, which in turn causes a loss in the mitochondrial membrane potential and an increase in the permeability of the mitochondrial outer membrane [45]. JC-1 was applied as a fluorescence probe in tumor cells to reflect the change of mitochondrial membrane potential. Cells after different treatments were shown in Fig. 3c, compared to the control group, tumor cells treated with 3BP@PLGA group and 3BP@PLGA-IR780 showed significantly weak red fluorescence and strong green signals. It indicated a pronounced decline in mitochondrial membrane potential. In the meantime, 3BP can also perturb mitochondria-associated OXPHOS, which is predominant for O_2_ consumption, by mainly inhibiting the activity of succinate dehydrogenase (SDH) on Complex II of mitochondria [20, 21, 44]. The disfunction of mitochondrial respiratory chain can lead to a plummeting of oxygen consumption. Oxygen electrode was employed to detect the oxygen consumption of 4T1 cells as a function of time (Fig. 3d). Results showed that compared to the control group, 3BP@PLGA reduced oxygen consumption of tumor cells, while 3BP@PLGA-IR780 achieved better inhibition effects (Fig. 3e).

The binding of HK-II to mitochondria plays an important role in regulating glucose metabolism and anti-apoptotic [23, 28]. Both the blockade of HK-II and the interference of mitochondrial respiration can lead to severe mitochondrial dysfunction, and further induce the apoptosis of the mitochondrial pathway [30, 45]. The Western blot results are shown in Fig. 3f. It can be seen that apoptotic correlation factors including Cytc and Caspase-3 showed high expression. The result also displayed significantly elevated the ratio of Bax/Bcl-2 at the mRNA level (Fig. 3f). The results further confirmed that 3BP activated the mitochondrial apoptotic signaling pathway. Based on the in vitro assessments, it is undoubtedly that 3BP showed reliable and efficient anti-tumor effects by acting on mitochondria. It is still noteworthy that the addition of IR780 tremendously reinforced the inhibitory effects of 3BP on HK-II and mitochondrial respiratory via endowing 3BP@PLGA-IR780 with the mitochondrial-targeting capability and offering 3BP a valuable shortcut to act on its most important target, further enabling a synergistic anti-tumor activity.

### Intracellular ROS generation and in vitro synergistic therapeutic effects of 3BP@PLGA-IR780

**Fig. 4:**
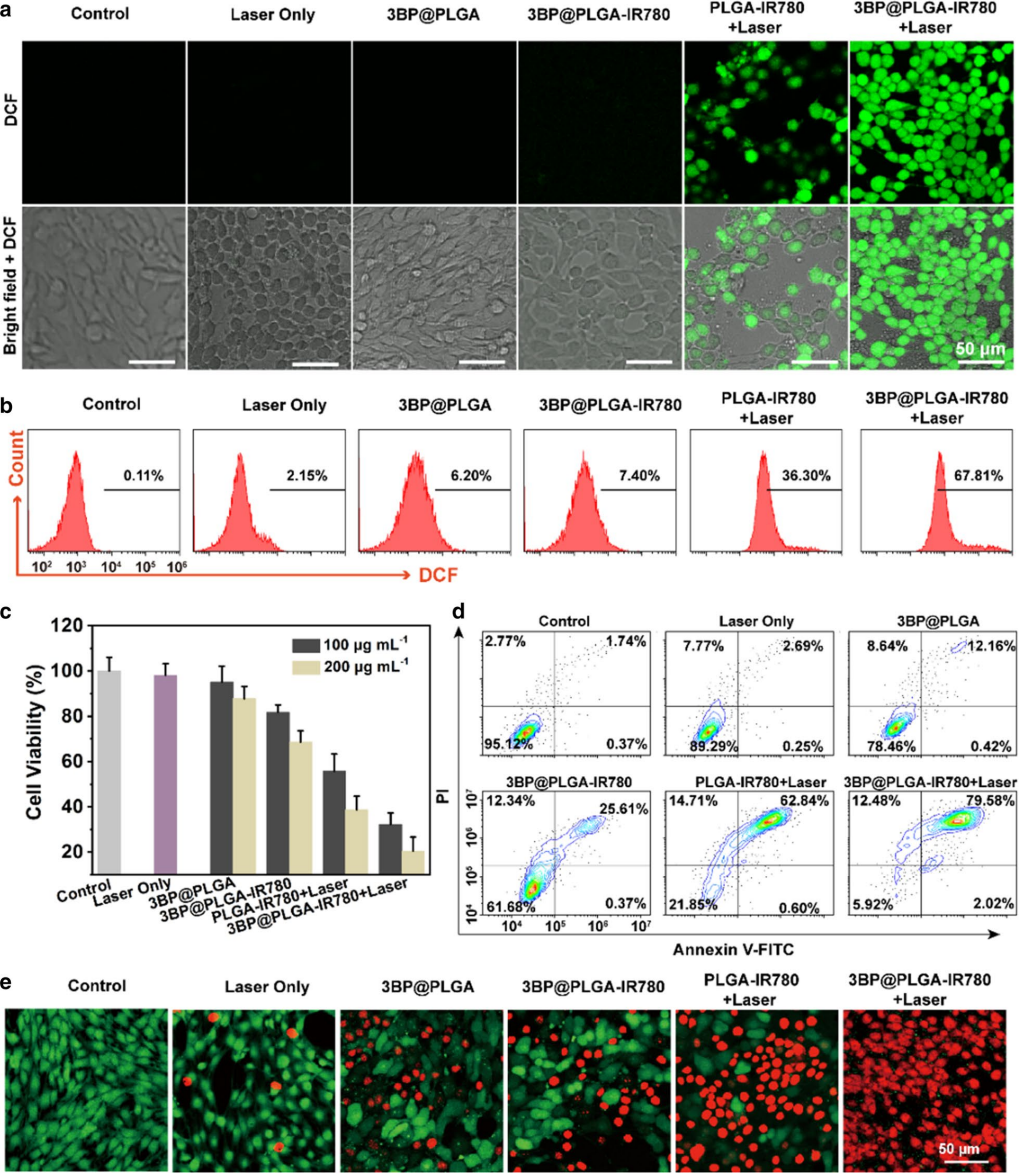
**a** Intracellular ROS generation after different treatments visualized by CLSM. **b** Flow cytometry analysis of ROS generation after different treatments. **c** Cell viabilities of 4T1 cells after various treatments. **d** Flow cytometry analysis of apoptosis rates after receiving different treatments. **e** CLSM images of calcein AM/PI co-stained 4T1 cells after various treatments (live and dead cells are stained green and red, respectively)

Given the encouraging ROS generation of 3BP@PLGA-IR780 in the aqueous solution, the ROS generation at the cellular level was further evaluated with a molecular probe DCFH-DA. After NIR laser irradiation (λ = 808 nm, 1.0 W cm^−2^) for 5 min, 4T1 cells in the PLGA-IR780 + Laser group emitted bright green fluorescence, which represented the consequential production of ROS (Fig. 4a). In addition, the fluorescence intensity in 3BP@PLGA-IR780 + Laser group was higher than that of PLGA-IR780 + Laser group, which resulted from the indispensable contribution of 3BP in relieving tumor hypoxia. Thus, this unique advantage of 3BP is expected to augment PDT efficacy. The quantitative results measured by flow cytometry were similar to CLSM observation that the strongest fluorescence intensity (67.81%) appeared in the group of 3BP@PLGA-IR780 + Laser (Fig. 4b). In contrast, almost no obvious fluorescence was observed in the groups of Laser Only, 3BP@PLGA and 3BP@PLGA-IR780 (2.15%, 6.20% and 7.40%, respectively). Such difference revealed that the assistance of 3BP in 3BP@PLGA-IR780 considerably improved the production of ROS in tumor cells.

Following the investigation of intracellular uptake and ROS generation, in vitro cytotoxicity of 3BP@PLGA-IR780 enhanced PDT against 4T1 cells was determined by a typical CCK-8 assay. As illustrated in Fig. 4c, a noticeable cell viability drop was observed after the treatments of 3BP@PLGA-IR780 + Laser and PLGA-IR780 + Laser at equivalent PLGA concentrations of 100 μg mL^−1^ and 200 μg mL^−1^. Particularly, the cytotoxicity of 3BP@PLGA-IR780 + Laser was higher than that of PLGA-IR780 + Laser at equivalent concentrations of IR780, probably indicating the synergistic therapeutic effects of 3BP-induced mitochondrial respiratory depression and IR780-based PDT. It is worth mentioning that 3BP could also perturb tumor metabolism via inhibition of HK-II and further induce reduction of ATP [23], which might make tumor cells more vulnerable to PDT damage [25, 26]. According to the in vitro assessments, compared to the control group, after 4 h of incubation with 3BP@PLGA and 3BP@PLGA-IR780, 4T1 cells presented loss of intracellular ATP, and 3BP@PLGA-IR780 led to a more significant ATP decline (Additional file 1: Fig. S7). These metabolic interruptions subsequently led to strong cytocidal activities on tumor cells. Moreover, the mitochondrial-targeting ability of IR780 would be expected to enhance the intracellular uptake and mitochondrial accumulation of 3BP. As shown in the absence of laser irradiation, 3BP@PLGA-IR780 still displayed more significant cell damage than 3BP@PLGA. Furthermore, flow cytometry analysis presented a similar trend in cellular damage (Fig. 4d). For instance, while assisted by laser irradiation, with equivalent PLGA concentrations at 200 μg mL^−1^, PLGA-IR780 + Laser induced approximately 60% cell viability decrease, whereas, with 3BP@PLGA-IR780 + Laser, the cell viability remarkably dropped about 80%. In addition, the cell viability was visualized by CLSM. After various treatments, 4T1 cells were stained with calcein-AM and PI to identify the living and dead cells. The CLSM images displayed that almost all cells presented red fluorescence in the 3BP@PLGA-IR780 + Laser group, indicating that severe cell apoptosis/necrosis occurred, which exhibited a effective cell killing ability (Fig. 4e).

The plausible mechanism for cytotoxicity might be explained as follows. On the one hand, 3BP could interfere mitochondria respiration chain by alkylating SDH on the complex II, causing an obvious decline in O_2_ consumption [22, 46]. The ameliorating of hypoxia further improved ROS generation directly. Meanwhile, the vulnerability of tumor cells due to the insufficient energy supply caused by HK-II inhibition cooperatively amplified the cell killing effects of PDT. On the other hand, the mitochondrial-targeting capability of IR780 enabled a large number of 3BP@PLGA-IR780 accommodating and functioning in tumor tissue, especially in mitochondria, which makes ROS more lethal to cells. Consequently, the synergy of 3BP and IR780 collaboratively enhanced the overall therapeutic efficacy.

### In vivo biodistribution (FL Imaging) and pharmacokinetics study of 3BP@PLGA-IR780

**Fig. 5:**
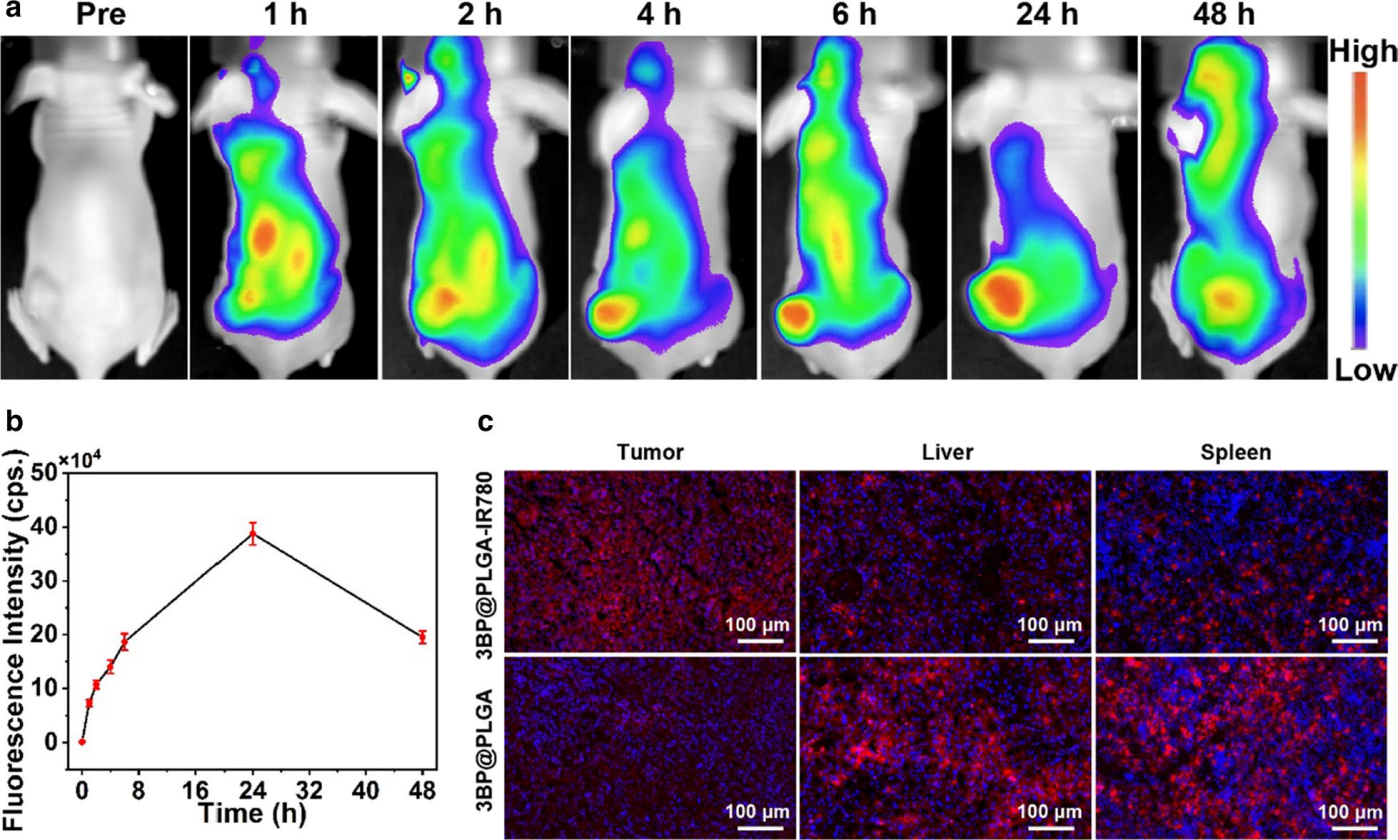
**a** In vivo FL imaging of 4T1 tumor-bearing mice after intravenous injection of 3BP@PLGA-IR780. **b** The corresponding FL intensity within tumor region with prolonged circulation time. **c** The biodistribution of 3BP@PLGA-IR780 in tumor region, liver and spleen (scale bar is 100 μm)

The efficient accumulation of 3BP@PLGA-IR780 in tumor tissue is a prerequisite for the following in vivo performance [47]. Fortunately, IR780 can selectively accumulate in tumor cells/tissues and emit fluorescence signals [48]. FL imaging was performed using 3BP@PLGA-IR780 as a contrast agent to detect the in vivo biodistribution. 4T1 tumor models were intravenously administrated with 3BP@PLGA-IR780 suspension. As depicted in Fig. 5a, FL images were collected at pre-injection, 1, 2, 4, 6, 24, and 48 h post-injection. It can be seen that the FL signal in the tumor site strengthened over time with a peak at 24 h (Fig. 5b). In addition to in vivo FL imaging, pathological examinations of major organs were also performed to further determine the biodistribution of 3BP@PLGA-IR780 in vivo. The tumor tissue and major organs were harvested after 24 h post-injection of 3BP@PLGA-IR780. The data revealed a remarkable accumulation of 3BP@PLGA-IR780 in the tumor site, where the fluorescence signals were higher than that of the liver and spleen (Fig. 5c). In comparison, in the 3BP@PLGA-treated group, the fluorescence signals extensively accumulated in the liver and spleen due to the phagocytosis effect of the mononuclear phagocytic system. These results showed that the unique tumor-targeting capability of IR780 endowed 3BP@PLGA-IR780 with efficient accumulation in the tumor sites. It indicates the enormous potential in selectively eradicating tumor cells and circumventing the systemic adverse effects.

The blood metabolism parameter and targeted organ distribution of the intravenously injected drugs are the primary research content of pharmacokinetics. In this study, the amounts of IR780 in blood of the healthy Balb/c nude mice and 4T1 tumor-bearing mice were assessed by microplate reader after intravenously injected with 3BP@PLGA-IR780. It was found that the level of 3BP@PLGA-IR780 in blood dropped dramatically at first, but tended to stabilize after 24 h of injection. By plotting the blood circulation time against drug concentration, the blood half-life of the drug in the tumor-bearing mice was calculated to be 8.72 h, which was slightly longer than 8.21 h of the heathy mice (Additional file 1: Fig. S8a and S8c). A potential possibility of half-life extension lies in the efficient accumulation of 3BP@PLGA-IR780 in tumor regions. With the extension of time, drugs in the blood circulation were gradually cleared. However, the tumor site, which might serve as a reservoir, accommodates a large amount of drugs at first and those drugs might gradually returned to the blood circulation later, contributing to the prolonged circulation time of 3BP@PLGA-IR780. Following the two-compartment pharmacokinetic model, the clearance rates of 3BP@PLGA-IR780 in healthy mice were calculated to be 0.17942 ± 0.03141 μg mL^−1^ h^−1^ in the first stage and it quickly decreased to 0.02263 ± 0.0013 μg mL^−1^ h^−1^ in the second stage. Similarly, the clearance rates of 3BP@PLGA-IR780 in tumor-bearing mice were 0.20205 ± 0.0404 μg mL^−1^ h^−1^ in the first stage and 0.0203 ± 0.0035 μg mL^−1^ h^−1^ in the second stage (Additional file 1: Fig. S8b and S8d). In addition, the ex vivo fluorescence imaging was employed to investigate the metabolism of 3BP@PLGA-IR780 in tumor tissues with prolonged administration time. The results showed that a large number of 3BP@PLGA-IR780 were enriched in tumor tissues one day after injection, maintained a high concentration on the 3rd day, and then presented a downward trend (Additional file 1: Fig. S9). On the 10th day, there was a negligible fluorescence signal in tumor tissues, suggesting that 3BP@PLGA-IR780 have been metabolized from tumor tissues. The above results confirmed that 3BP@PLGA-IR780 had excellent pharmacokinetic behavior, including long blood circulation time, high tumor enrichment, and rapid metabolic behavior in vivo.

### Deep penetration of 3BP@PLGA-IR780

**Fig. 6:**
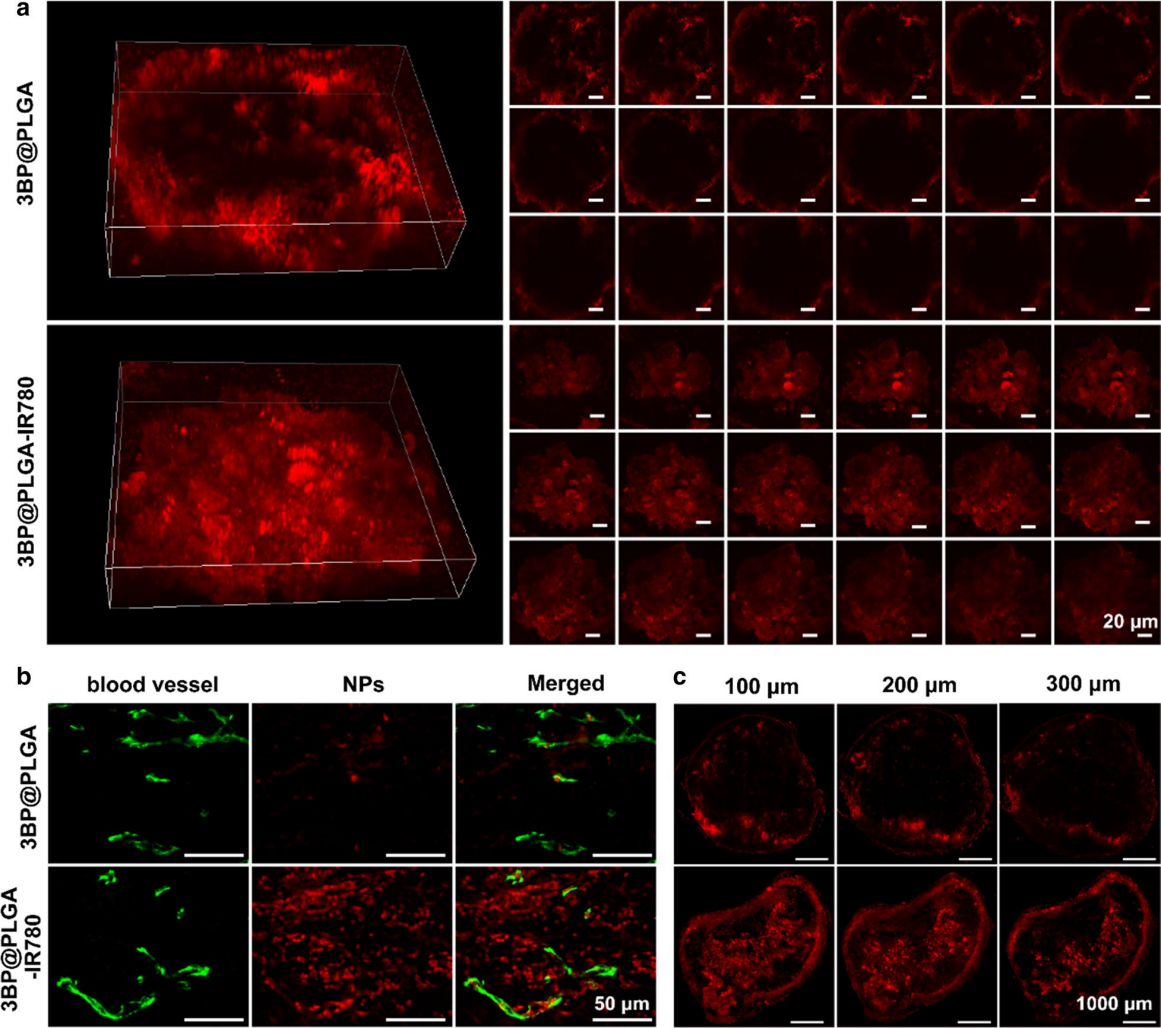
**a** Intertumoral diffusion of 3BP@PLGA or 3BP@PLGA-IR780 in 3D tumor models (scale bar is 20 μm). **b** The distribution of 3BP@PLGA or 3BP@PLGA-IR780 around tumor vessels (scale bar is 50 μm). **c** Distribution of nanoplatforms in different tumor sections (the interval is 100 μm)

Nanomedicine is of great significance in tumor regression on account of its biocompatibility and reduced toxicity. However, manufacturing nanomedicine that allows tumor accumulation and deep diffusion inside solid tumor needs continuous optimization [49, 50]. Taken into account the complicacy of the tumor microenvironment, 3D tumor spheroid was employed to mimic the in vivo solid tumor as it could offer near-identical tumor microenvironmental characteristics [51, 52]. As shown in Fig. 6a, red fluorescence originating from Dil-labeled 3BP@PLGA-IR780 was found to diffuse throughout the tumor spheroid, while 3BP@PLGA was found only distributed around the peripheral areas, suggesting that IR780 endowed the nanoplatforms desirable capability to penetrate deep inside 4T1 spheroids more efficiently. In addition, we further explored the distribution of nanoplatforms around the tumor vessels. The vasculature was stained by CD31 antibody (green fluorescence), and the nanoplatform was labeled by Dil (Fig. 6b). It was found that substantial 3BP@PLGA-IR780 nanoplatforms distributed farther away from the blood vessels and spread over tumor tissue, while 3BP@PLGA mainly gathered inside or only adjacent to tumor vessels. The solid tumor tissues were then sliced for further observation. As shown in Fig. 6c, the red fluorescence of 3BP@PLGA only accumulated around the surface of tumor tissue, while 3BP@PLGA-IR780 could widely distribute deep inside the tumor. It again confirmed the effective penetration of it.

### In vitro and in vivo PA imaging of 3BP@PLGA-IR780

**Fig. 7:**
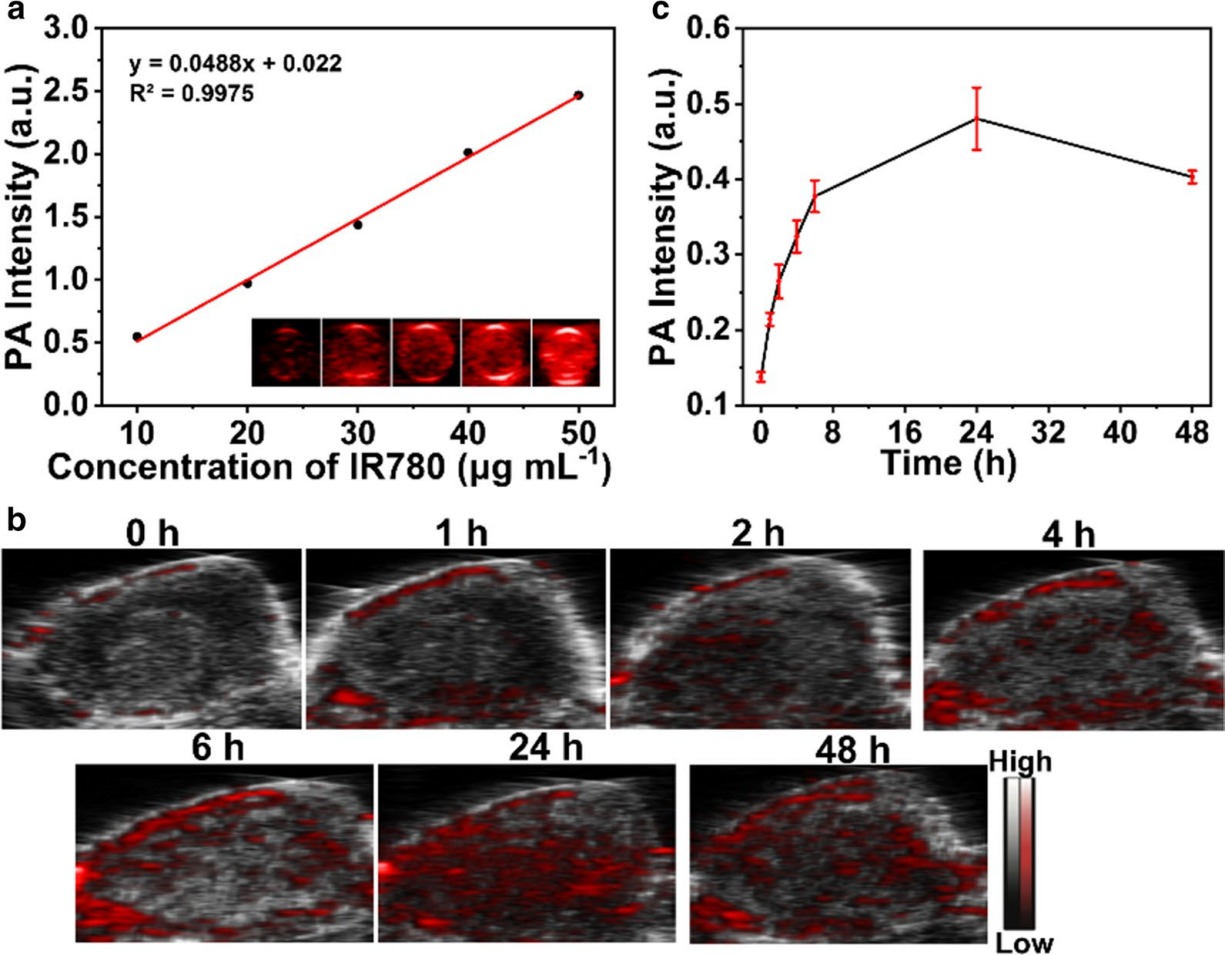
**a** Concentration-dependent PA intensity of 3BP@PLGA-IR780. **b** In vivo PA images of 4T1 tumor tissue with prolonged injection time (0, 1, 2, 4, 6, 24, and 48 h). **c** The PA intensity data of tumor tissues at different time points

Probes with imaging guidance ability have great practical benefits for enhancing the accuracy of anticancer therapy [53]. On account of the unique absorbance in NIR region, IR780 was considered to be an eligible PA contrast agent. PA imaging capability of 3BP@PLGA-IR780 nanoplatforms was tested both in vitro and in vivo. For PA laser excitation wavelength from 680 to 970 nm (interval = 5 nm), 3BP@PLGA-IR780 showed an optimal wavelength at 785 nm (Additional file 1: Fig. S10). Meanwhile, the PA signals increased linearly with an elevated concentration of IR780 from 10 to 50 µg mL^−1^ (Fig. 7a). For in vivo evaluation, 4T1-xenograft tumors were intravenously injected with 3BP@PLGA-IR780 suspension. As shown in Fig. 7b, an obvious PA signal highlighted the tumor region, strengthened over time, and reached a maximum value at 24 h post-injection (Fig. 7c). The results confirmed the reliable imaging capability of 3BP@PLGA-IR780 to differentiate targeted regions from normal tissues.

### Alleviation of tumor hypoxia assisted by 3BP@PLGA-IR780

**Fig. 8:**
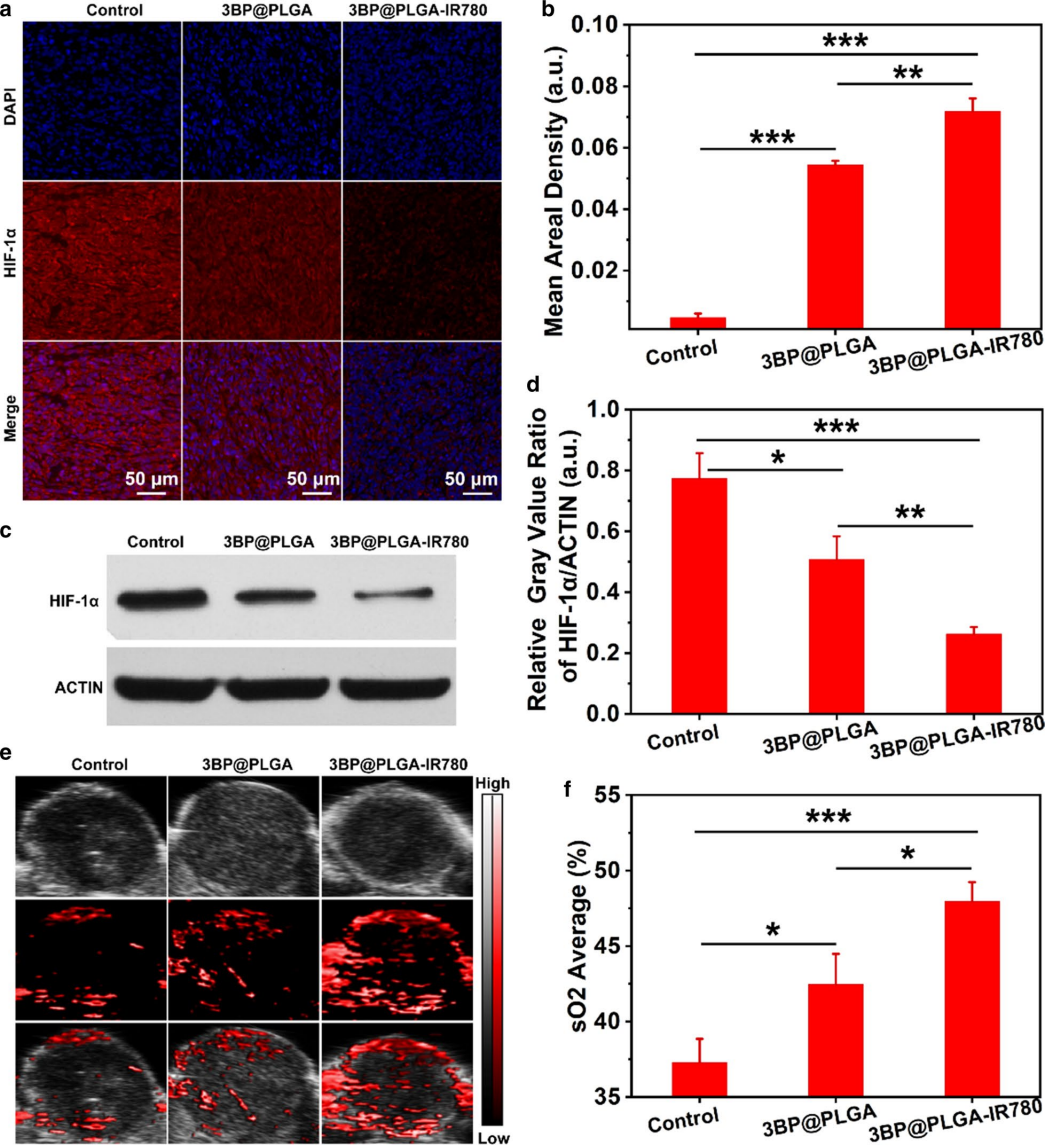
**a** Immunofluorescent images of 4T1 tumor slices stained by HIF-1α. **b** The corresponding quantitative analysis of HIF-1α fluorescence intensities. **c** Western blot results of HIF-1α expression in tumors treated with 3BP@PLGA or 3BP@PLGA-IR780. **d** The corresponding quantitative analysis of HIF-1α after different treatments. **e** Oxyhemoglobin saturation monitoring after injection of 3BP@PLGA or 3BP@PLGA-IR780. **f** Quantitative analysis of sO_2_ within tumor regions

It has been reported that 3BP is capable of overcoming tumor hypoxia as it was able to suppress oxygen consumption of tumors via interfering with mitochondria-associated OXPHOS [44]. To verify this hypothesis, immunofluorescence of HIF-1α, the corresponding western blot assay and the quantifying of oxygenated hemoglobin with PA imaging system were conducted after various treatments. As shown in Fig. 8a and b, the expression of HIF-1α was relatively high in the control group, implying that the tumor was in a hypoxia state. Comparatively, tumor hypoxia was greatly relieved after the treatment of 3BP@PLGA-IR780, as indicated by the weak fluorescence in the tumor. Moreover, the results of western blot were consistent with immunofluorescence staining, demonstrating that 3BP@PLGA-IR780 induced a significant decrease in the expression of HIF-1α (Fig. 8c and d). In particular, with the addition of IR780, the expression of HIF-1α in tumor tissues treated with 3BP@PLGA-IR780 was drastically less than that in cells treated with 3BP@PLGA, indicating that the tumor-targeted properties of IR780 endowed 3BP@PLGA-IR780 with more effective accumulation in tumor sites. Similarly, as shown in Fig. 8e and f, the oxyhemoglobin signal significantly increased and diffused around the tumor region after injection of 3BP@PLGA-IR780 compared to the control group, while 3BP@PLGA exhibited a slightly inferior performance. These results revealed that 3BP loaded in 3BP@PLGA had a noticeable yet limited capability of alleviating hypoxia in the tumor area. But when in combination with IR780, 3BP@PLGA-IR780 was able to achieve more effective alleviation of tumor hypoxia, which is also expected to ensure the efficiency of PDT.

### In vivo synergistic tumor therapy

**Fig. 9:**
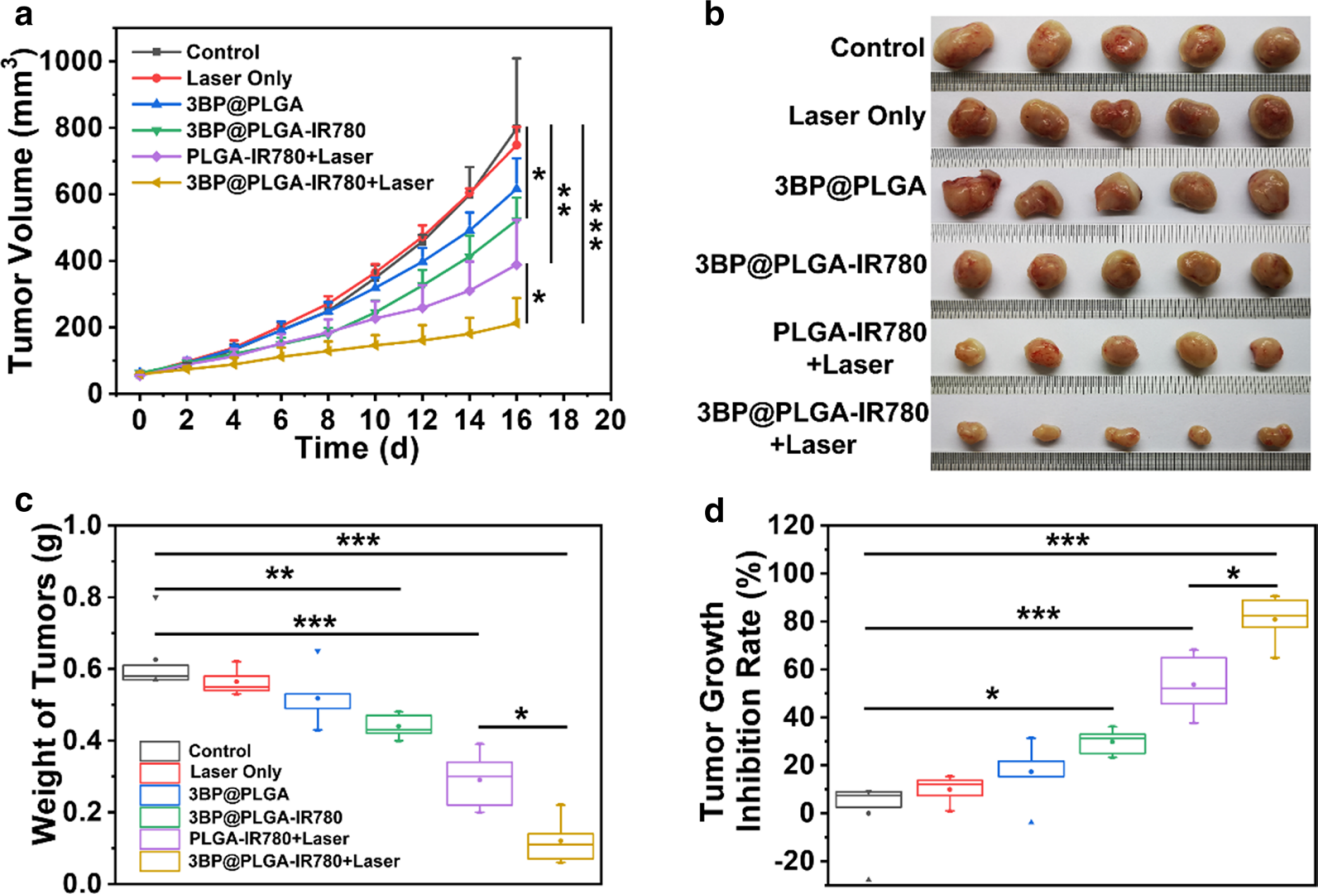
**a** Tumor growth curves of mice after various treatments. **b** Digital photographs of tumor nodes dissected from mice in six groups after kinds of treatments. **c** Weight of tumor tissues in each group at the end of treatment. **d** Tumor inhibition rate of mice after different treatments

After confirmation of the mitochondria-targeting capability, deep penetration, and hypoxia relieve of 3BP@PLGA-IR780, the in vivo synergistic tumor therapy was further assessed. Bearing these properties in mind, 4T1 tumor-bearing mice were randomly divided into six groups (n = 5): (1) Control (Saline); (2) Laser only; (3) 3BP@PLGA; (4) 3BP@PLGA-IR780; (5) PLGA-IR780 + Laser; (6) 3BP@PLGA-IR780 + Laser. When the tumor volume reached a size of 50–70 mm^3^, mice were intravenously injected with saline, 3BP@PLGA, PLGA-IR780 or 3BP@PLGA-IR780 suspensions. For PLGA-IR780 + Laser and 3BP@PLGA-IR780 + Laser groups, NIR laser irradiation (1.0 W cm^−2^) would be conducted 24 h after injection. To exclude the photothermal effect, the laser irradiation was implemented for 40 s on and 40 s off to keep the tumor temperature under 42 °C. The above irradiation was repeated for 15 cycles (Additional file 1: Fig. S11). The tumor volumes of mice were monitored every other day during the therapeutic period (Fig. 9a), and the tumor tissues were excised and weighted at the end of the treatments (Fig. 9b and c). The weights of tumors resected from the mice in each group presented a similar trend as the tumor volumes.

As a result, the tumors in the control group and laser only group grew steadily. In contrast, the tumor growth of mice treated with 3BP@PLGA and 3BP@PLGA-IR780 were slightly suppressed, which might be due the therapeutic effects induced by 3BP, such as metabolic disorder and activation of apoptosis (Fig. 9d). Comparatively, obvious tumor growth inhibitions were observed both in the PLGA-IR780 + Laser group and 3BP@PLGA-IR780 + Laser group, implicating the outstanding therapeutic efficacy of PDT treatment. Importantly, the PLGA-IR780 + Laser group induced about 53.67% inhibition rate, while the mice receiving 3BP@PLGA-IR780 + Laser therapy exhibited the highest tumor suppression (80.83 ± 10.29%), indicating that the synergy of 3BP and IR780 undeniably favored PDT effects.

**Fig. 10:**
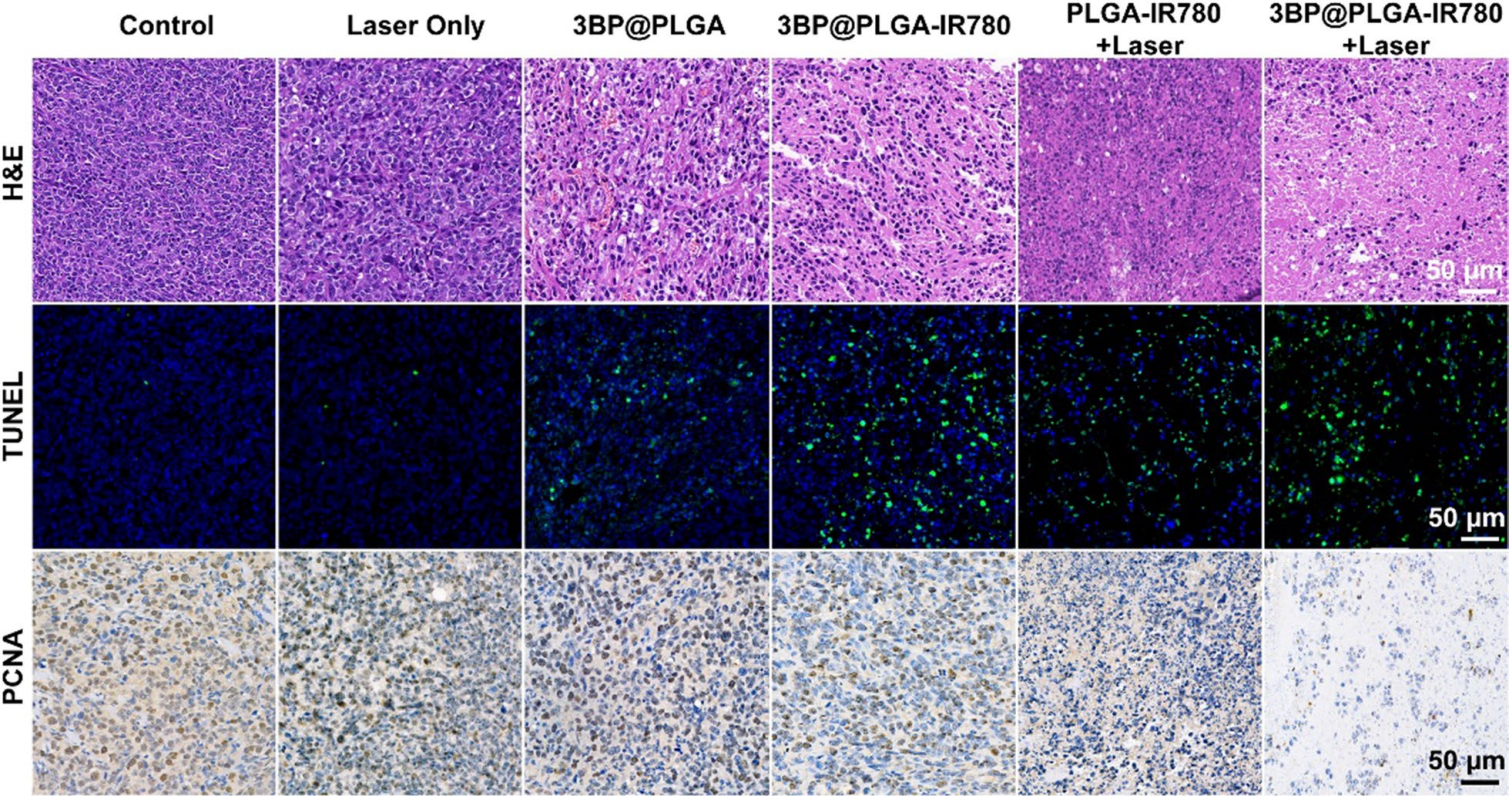
H&E, TUNEL and PCNA staining of tumors after different treatments

Furthermore, H&E, TUNEL, and PCNA staining on tumor sections were conducted to confirm the synergistic and amplified PDT effects (Fig. 10). As shown in H&E and TUNEL-stained tumor tissues, almost all cells suffered from severe apoptosis/necrosis in the 3BP@PLGA-IR780 group. The PCNA staining of tumor tissues followed a similar tendency and presented a significantly lower proliferation index in the 3BP@PLGA-IR780 + Laser group.

### Biosafety assessment of 3BP@PLGA-IR780

The relative weights of mice were measured during the treatment period, which showed negligible changes (Additional file 1: Fig. S12). H&E staining of the main organs was performed at the end of various treatments, and no obvious histopathological lesion was observed (Additional file 1: Fig. S13). The above results indicated the satisfactory biosafety of 3BP@PLGA-IR780-mediated therapy. To further determine the in vivo biocompatibility 3BP@PLGA-IR780, blood cell analysis and biochemical examination of blood were conducted in healthy mice after intravenous administration with 3BP@PLGA-IR780. The results of blood cell analysis and biochemical examination showed no obvious abnormality in the indicators of hepatotoxicity, nephrotoxicity, and blood cell count (Additional file 1: Fig. S14a). In addition, the main organs (heart, liver, spleen, lung, and kidney) of the mice were harvested at different time points (7, 15, 30 days). There was no histopathological lesion found in these organs (Additional file 1: Fig. S14b), showing rare systemic toxicity to mice at the treatment dose and confirming the high biocompatibility of 3BP@PLGA-IR780.

## Discussion

Hypoxia in tumor sites greatly limits the yield of ROS and further weakens the therapeutic effect of PDT [5, 7]. Although some strategies were designed to directly deliver oxygen molecules or hydrogen peroxide catalysts towards tumor sites in many previous studies [10, 13, 14, 54], there are still deficiencies, such as limited oxygen loading, premature oxygen release, and low efficiency of oxygen production [15]. This study focuses on the respiration of tumor mitochondria, which accounts for the predominant oxygen consumption, attempting to reduce the oxygen consumption of tumor cells by inhibiting the mitochondrial respiratory chain. It can remedy the shortcoming of limited loading capacity to some extent. In addition, as mitochondria plays a pivotal role in cell survival, therapeutic modality with mitochondria as subcellular targets can amplify the lethal effects on tumor cells [29]. 3BP was introduced in this multifunctional nanoplatform to perturb mitochondria-associated OXPHOS by mainly inhibiting the activity of SDH on Complex II of mitochondria [20, 21, 44]. The detection results of oxygen electrode showed that the dissolved O_2_ in culture medium of tumor cells treated by 3BP@PLGA was much higher than that of the control. Likewise, significant increases in oxyhemoglobin signal within tumor tissue were monitored by a PA imaging after treatment. Moreover, the expression of HIF-1α of tumor tissues significantly decreased. It is still noteworthy that the addition of IR780 tremendously reinforced the inhibitory effects of 3BP on mitochondrial respiratory. Correspondingly, encouraging increases of ROS generation after PDT were observed in both CLSM and flow cytometry.

IR780 was introduced as the PS. Compared to typical indocyanine green (ICG), IR780 shows not only higher fluorescence intensity [55, 56], but also has a much higher ^1^O_2_ quantum yield [57, 58], which indicates a relatively stronger therapeutic effect at the equivalent drug concentration. Indeed, the hydrophobicity of IR780 could lead to poor distribution, which limits its clinical application. However, its lipophilic property allows it to be efficiently loaded in the shell layer of PLGA nanoplatform with good biocompatibility, which overcomes the difficulty of clinical administration [59]. IR780 was dissolved in CHCl_2_ during the synthesis of 3BP@PLGA-IR780 and embedded in PLGA shell, which could protect the IR780 from spontaneous quenching. This makes IR780 more tenacious in biological environments and enhanced the colloidal stability of IR780. In particular, IR780 shows inherent characteristics of preferential accumulation in some specific tumor cells. Organic anion transit peptide (OATP) plays a predominant role in the cellular uptake of IR780, which has been proved to contribute to the tumor-targeting behavior of IR780 [34]. In addition, the special status of glycolysis and plasma membrane potential in tumor cells also benefit the tumor preferential accumulation of IR780 [37]. Therefore, the tumor targeting ability of IR780 provided 3BP@PLGA-IR780 a preferential access to enter tumor cells, presenting a stronger fluorescence intensity than 3BP@PLGA in the intracellular uptake detection. As mitochondria are vulnerable to ROS damage, PDT with mitochondria as subcellular targets can amplify the lethal effects of ROS on tumor cells [60, 61]. At present, delivery of compounds to mitochondria by attaching various mitochondria-targeting lipophilic cations, such as triphenyl phosphonium (TPP +), rhodamine, cyanine derivatives, and cationic peptides, have gained much traction [62]. In particular, due to its heptamethine core with lipophilic cationic property and the high membrane potential of tumor mitochondria, IR780 can bind to the mitochondria proteins of tumor cells and be retained in the mitochondria [34, 36]. Hence, IR780 endowed 3BP@PLGA-IR780 with the inherent mitochondrial-targeting capability, and offered 3BP a valuable shortcut to act on its most important target, mitochondrial HK-2 [23]. The reduction in HK-II expression and ATP production both demonstrated remarkable increases in the inhibition effects of 3BP@PLGA-IR780 on HK-II. Enhanced blockade to HK-II subsequently led to severe mitochondrial dysfunction and activation of the mitochondrial apoptotic pathway [30, 31]. As a result, pronounced decline in mitochondrial membrane potential and high expression of the factors associated to the mitochondrial apoptosis pathway were observed after 3BP@PLGA-IR780 treatment. Consequently, the lethal damages from multiple ways collaboratively enhanced the overall therapeutic efficacy. Supported by the results of CCK8, 3BP@PLGA-IR780 achieved greater improvement in the tumor cell killing performance than 3BP@PLGA.

Generally, most nanocarriers enter the tumor site via the enhanced permeability and retention (EPR) effect [63], but it’s difficult for them to penetrate deeper into the tumor tissues due to the complex tumor microenvironment (e.g., solid extracellular matrix, tight cell packing density and high interstitial flow pressure) [64], which further attenuates the therapeutic efficacy. Therefore, a variety of strategies have been reported to promote the permeation of nanocarriers including synthesis of small size or size shrinkable nanocarriers [65] and assembling nanocarriers with the tumor-penetrating cyclic peptide (e.g., iRGD) [64]. In this study, 3D tumor spheroid models were employed to assess the penetration ability of 3BP@PLGA-IR780 in vitro. 3BP@PLGA-IR780 was found to diffuse throughout the tumor spheroid, presenting a more even distribution. In addition, the in vivo experiment also proved that 3BP@PLGA-IR780 could distribute farther away from the blood vessels and spread over the tumor tissue. OATP-mediated transporting between tumor cells may contribute to the deep penetration activity of IR780 [66]. This desirable deep penetration ability could compensate for the inhomogeneous distribution of general nanocarriers, facilitating drug delivery and therapeutic efficacy. 3BP@PLGA-IR780 nanoplatforms also exhibited excellent pharmacokinetic behaviors including long blood circulation time and rapid metabolic behavior in vivo. 3BP@PLGA-IR780 nanoplatforms maintained a high concentration in tumor regions from 24 h after injection to the 3rd day, which provided a long enough therapeutic time window. In addition, based on fluorescence imaging and PA imaging, the multimodal imaging capability of 3BP@PLGA-IR780 nanoplatforms will facilitate monitoring the distribution of 3BP@PLGA-IR780 in the tumor site and improve treatment precision.

## Conclusion

In conclusion, multifunctional 3BP@PLGA-IR780 nanoplatform was synthesized for highly efficient PDT and a series of synergy to strengthen the therapeutic effects. The nanoplatform has been demonstrated to alleviate tumor hypoxia by decreasing physiological O_2_ consumption. Besides, 3BP@PLGA-IR780 could interrupt the energy metabolism of tumor cells. Moreover, this nanoplatform showed tumor-targeting, mitochondria-targeting and deep tumor penetration capabilities, which was favorable to therapy delivery. Moreover, PA/FL dual-modal imaging capability allowed real-time guidance and improved precision for PDT. In addition, the desirable biosafety of 3BP@PLGA-IR780 nanoplatform ensured the feasibility towards clinical transformation. This study provides an ideal strategy for cancer therapy by concurrent oxygen consumption reduction, oxygen-augmented PDT, energy supply decrease, mitochondria-targeted/deep-penetrated nanoplatforms and PA/FL dual-modal imaging guidance/monitoring.

## Supplementary Information

**Additional file 1:** **Fig. S1.** Characterizations of 3BP@PLGA. **Fig. S2.** (a) UV–vis-NIR absorbance spectra of IR780 at elevated concentrations. (b) The relative absorbance of IR780 in the UV–vis spectrum at 789 nm. **Fig. S3.** (a) UV–vis-NIR absorbance spectra of 3BP at elevated concentrations. (b) The relative absorbance of 3BP in the UV–vis spectrum at 326 nm. **Fig. S4.** UV–vis-NIR absorbance spectra of 3BP@PLGA-IR780 and 3BP@PLGA. **Fig. S5.** Photo-stability study of 3BP@PLGA-IR780. **Fig. S6.** CLSM images of intracellular uptake of 3BP@PLGA-IR780 and 3BP@PLGA for MCF-7 and MBA-MD-231 cells. **Fig. S7.** (a) ATP standard curve and (b) ATP concentrations after different treatments. **Fig. S8.** Pharmacokinetics Study of 3BP@PLGA-IR780. **Fig. S9.** Ex vivo fluorescence imaging pictures of isolated tumors at 1, 3, 5, 7 and 10 days after intravenous injection of 3BP@PLGA-IR780. **Fig. S10.** PA signal changes of 3BP@PLGA-IR780 with PA laser excitation wavelengths ranging from 680 to 970 nm. **Fig. S11.** Representative tumor temperature change curve when receiving PDT (1.0 W cm^−2^, on 40 s, off to room temperature, 15 cycles). **Fig. S12.** Body-weight curves of six groups after various treatments. **Fig. S13.** H&E staining of the major organs of 4T1 tumor-bearing mice after different treatments. **Fig. S14.** (a) Hematological and blood biochemistry analysis of healthy mice after intravenous injection of 3BP@PLGA-IR780. (b) H&E staining of major organs of mice post injection of 3BP@PLGA-IR780.

## Data Availability

All data analyzed during this study are included in this published article.

## References

[1] Li X, Lovell JF, Yoon J, Chen X (2020). Clinical development and potential of photothermal and photodynamic therapies for cancer. Nat Rev Clin Oncol.

[2] Xie J, Wang Y, Choi W, Jangili P, Ge Y, Xu Y, Kang J, Liu L, Zhang B, Xie Z (2021). Overcoming barriers in photodynamic therapy harnessing nano-formulation strategies. Chem Soc Rev.

[3] Zhao X, Liu J, Fan J, Chao H, Peng X (2021). Recent progress in photosensitizers for overcoming the challenges of photodynamic therapy: from molecular design to application. Chem Soc Rev.

[4] Zhou Z, Song J, Nie L, Chen X (2016). Reactive oxygen species generating systems meeting challenges of photodynamic cancer therapy. Chem Soc Rev.

[5] Sun Y, Zhao D, Wang G, Wang Y, Cao L, Sun J, Jiang Q, He Z (2020). Recent progress of hypoxia-modulated multifunctional nanomedicines to enhance photodynamic therapy: opportunities, challenges, and future development. Acta pharmaceutica Sinica B.

[6] Liu JN, Bu W, Shi J (2017). Chemical design and synthesis of functionalized probes for imaging and treating tumor hypoxia. Chem Rev.

[7] Li X, Kwon N, Guo T, Liu Z, Yoon J (2018). Innovative strategies for hypoxic-tumor photodynamic therapy. Angew Chem Int Ed Engl.

[8] Barker HE, Paget JT, Khan AA, Harrington KJ (2015). The tumour microenvironment after radiotherapy: mechanisms of resistance and recurrence. Nat Rev Cancer.

[9] Jing X, Yang F, Shao C, Wei K, Xie M, Shen H, Shu Y (2019). Role of hypoxia in cancer therapy by regulating the tumor microenvironment. Mol Cancer.

[10] Zhang L, Wang D, Yang K, Sheng D, Tan B, Wang Z, Ran H, Yi H, Zhong Y, Lin H (2018). Mitochondria-targeted artificial “Nano-RBCs” for amplified synergistic cancer phototherapy by a single NIR irradiation. Adv Sci.

[11] Godet I, Shin YJ, Ju JA, Ye IC, Wang G, Gilkes DM (2019). Fate-mapping post-hypoxic tumor cells reveals a ROS-resistant phenotype that promotes metastasis. Nat Commun.

[12] Zhou TJ, Xing L, Fan YT, Cui PF, Jiang HL (2019). Light triggered oxygen-affording engines for repeated hypoxia-resistant photodynamic therapy. J Control Release.

[13] Liu WL, Liu T, Zou MZ, Yu WY, Li CX, He ZY, Zhang MK, Liu MD, Li ZH, Feng J (2018). Aggressive man-made red blood cells for hypoxia-resistant photodynamic therapy. Adv Mater.

[14] Wang J, Sun J, Hu W, Wang Y, Chou T, Zhang B, Zhang Q, Ren L, Wang H (2020). A Porous Au@Rh bimetallic core-shell nanostructure as an H_2_O_2_-driven oxygenerator to alleviate tumor hypoxia for simultaneous bimodal imaging and enhanced photodynamic therapy. Adv Mater.

[15] Yu W, Liu T, Zhang M, Wang Z, Ye J, Li CX, Liu W, Li R, Feng J, Zhang XZ (2019). O_2_ economizer for inhibiting cell respiration to combat the hypoxia obstacle in tumor treatments. ACS Nano.

[16] Hernansanz-Agustín P, Choya-Foces C, Carregal-Romero S, Ramos E, Oliva T, Villa-Piña T, Moreno L, Izquierdo-Álvarez A, Cabrera-García JD, Cortés A (2020). Na(+) controls hypoxic signalling by the mitochondrial respiratory chain. Nature.

[17] Yang Z, Wang J, Liu S, Li X, Miao L, Yang B, Zhang C, He J, Ai S, Guan W (2020). Defeating relapsed and refractory malignancies through a nano-enabled mitochondria-mediated respiratory inhibition and damage pathway. Biomaterials.

[18] Fan Y-T, Zhou T-J, Cui P-F, He Y-J, Chang X, Xing L, Jiang H-L. Modulation of intracellular oxygen pressure by dual-drug nanoparticles to enhance photodynamic therapy. Adv Funct Mater. 2019;29:1806708.

[19] El Sayed SM (2018). Enhancing anticancer effects, decreasing risks and solving practical problems facing 3-bromopyruvate in clinical oncology: 10 years of research experience. Int J Nanomedicine.

[20] Sobotka O, Endlicher R, Drahota Z, Kučera O, Rychtrmoc D, Raad M, Hakeem K, Červinková Z (2016). Impaired mitochondrial functions contribute to 3-bromopyruvate toxicity in primary rat and mouse hepatocytes. J Bioenerg Biomembr.

[21] Marrache S, Dhar S (2015). The energy blocker inside the power house: mitochondria targeted delivery of 3-bromopyruvate. Chem Sci.

[22] Rodrigues-Ferreira C, da Silva AP, Galina A (2012). Effect of the antitumoral alkylating agent 3-bromopyruvate on mitochondrial respiration: role of mitochondrially bound hexokinase. J Bioenerg Biomembr.

[23] Mathupala SP, Ko YH, Pedersen PL (2009). Hexokinase-2 bound to mitochondria: cancer’s stygian link to the “Warburg Effect” and a pivotal target for effective therapy. Semin Cancer Biol.

[24] Pereirada da Silva AP, El-Bacha T, Kyaw N, dos Santos RS, da Silva WS, Almeida FC, Da Poian AT, Galina A (2009). Inhibition of energy-producing pathways of HepG2 cells by 3-bromopyruvate. Biochem J.

[25] Dumas JF, Brisson L, Chevalier S, Mahéo K, Fromont G, Moussata D, Besson P, Roger S (2017). Metabolic reprogramming in cancer cells, consequences on pH and tumour progression: integrated therapeutic perspectives with dietary lipids as adjuvant to anticancer treatment. Semin Cancer Biol.

[26] Varghese E, Samuel SM, Líšková A, Samec M, Kubatka P, Büsselberg D. Targeting glucose metabolism to overcome resistance to anticancer chemotherapy in breast cancer. Cancers. 2020;12. 10.3390/cancers12082252 PMC7464784 32806533

[27] Krasnov GS, Dmitriev AA, Lakunina VA, Kirpiy AA, Kudryavtseva AV (2013). Targeting VDAC-bound hexokinase II: a promising approach for concomitant anti-cancer therapy. Expert Opin Ther Targets.

[28] Mathupala SP, Ko YH, Pedersen PL (2006). Hexokinase II: cancer’s double-edged sword acting as both facilitator and gatekeeper of malignancy when bound to mitochondria. Oncogene.

[29] Fulda S, Galluzzi L, Kroemer G (2010). Targeting mitochondria for cancer therapy. Nat Rev Drug Discov.

[30] Galina A (2014). Mitochondria: 3-bromopyruvate vs. mitochondria? A small molecule that attacks tumors by targeting their bioenergetic diversity. Int J Biochem Cell Biol.

[31] Wang TA, Zhang XD, Guo XY, Xian SL, Lu YF (2016). 3-bromopyruvate and sodium citrate target glycolysis, suppress survivin, and induce mitochondrial-mediated apoptosis in gastric cancer cells and inhibit gastric orthotopic transplantation tumor growth. Oncol Rep.

[32] Huang J, Zhang L, Zhou W, Wang J, Zhang R, Wang Z, Ran H, Li P, Li R (2021). Dual mitigation of immunosuppression combined with photothermal inhibition for highly effective primary tumor and metastases therapy. Biomaterials.

[33] Wang L, Niu C (2021). IR780-based nanomaterials for cancer imaging and therapy. J Mater Chem B.

[34] Zhang C, Liu T, Su Y, Luo S, Zhu Y, Tan X, Fan S, Zhang L, Zhou Y, Cheng T (2010). A near-infrared fluorescent heptamethine indocyanine dye with preferential tumor accumulation for in vivo imaging. Biomaterials.

[35] Zhang L, Yi H, Song J, Huang J, Yang K, Tan B, Wang D, Yang N, Wang Z, Li X (2019). Mitochondria-targeted and ultrasound-activated nanodroplets for enhanced deep-penetration sonodynamic cancer therapy. ACS Appl Mater Interfaces.

[36] Luo S, Tan X, Fang S, Wang Y, Liu T, Wang X, Yuan Y, Sun H, Qi Q, Shi C (2016). Mitochondria-targeted small-molecule fluorophores for dual modal cancer phototherapy. Adv Funct Mater.

[37] Zhang E, Luo S, Tan X, Shi C (2014). Mechanistic study of IR-780 dye as a potential tumor targeting and drug delivery agent. Biomaterials.

[38] Wang S, Wu KT, Xue DF, Zhang C, Rajput SA, Qi DS (2021). Mechanism of deoxynivalenol mediated gastrointestinal toxicity: insights from mitochondrial dysfunction. Food Chem Toxicol.

[39] Liu T, Han Y, Zhou T, Zhang R, Chen H, Chen S, Zhao H (2019). Mechanisms of ROS-induced mitochondria-dependent apoptosis underlying liquid storage of goat spermatozoa. Aging (Albany N Y).

[40] Song J, Zhang L, Yi H, Huang J, Zhang N, Zhong Y, Hao L, Ke Y, Wang Z, Wang D (2019). NIR-responsive nanoplatform for pre/intraoperative image-guided carcinoma surgery and photothermal ablation of residual tumor tissue. Nanomed Nanotechnol Biol Med.

[41] Kenry, Yeo T, Manghnani PN, Middha E, Pan Y, Chen H, Lim CT, Liu B (2020). Mechanistic understanding of the biological responses to polymeric nanoparticles. ACS Nano.

[42] Wang Y, Wang B, Zhang L, Huang J, Li P, Zhao Y, Zhou C, Liu M, Li W, He J (2020). Mitochondria-targeted nanospheres with deep tumor penetration for photo/starvation therapy. J Mater Chem B.

[43] Gandham SK, Talekar M, Singh A, Amiji MM (2015). Inhibition of hexokinase-2 with targeted liposomal 3-bromopyruvate in an ovarian tumor spheroid model of aerobic glycolysis. Int J Nanomedicine.

[44] Deng Y, Song P, Chen X, Huang Y, Hong L, Jin Q, Ji J (2020). 3-Bromopyruvate-conjugated nanoplatform-induced pro-death autophagy for enhanced photodynamic therapy against hypoxic tumor. ACS Nano.

[45] Gwak GY, Yoon JH, Kim KM, Lee HS, Chung JW, Gores GJ (2005). Hypoxia stimulates proliferation of human hepatoma cells through the induction of hexokinase II expression. J Hepatol.

[46] Pucelik B, Sułek A, Barzowska A, Dąbrowski JM (2020). Recent advances in strategies for overcoming hypoxia in photodynamic therapy of cancer. Cancer Lett.

[47] Lee YT, Tan YJ, Oon CE (2018). Molecular targeted therapy: treating cancer with specificity. Eur J Pharmacol.

[48] Potara M, Nagy-Simon T, Focsan M, Licarete E, Soritau O, Vulpoi A, Astilean S. Folate-targeted Pluronic-chitosan nanocapsules loaded with IR780 for near-infrared fluorescence imaging and photothermal-photodynamic therapy of ovarian cancer. Colloids Surf., B. 2021;203. 10.1016/j.colsurfb.2021.111755 33862575

[49] Minchinton AI, Tannock IF (2006). Drug penetration in solid tumours. Nat Rev Cancer.

[50] Sun Y (2016). Tumor microenvironment and cancer therapy resistance. Cancer Lett.

[51] Kim J, Jo C, Lim WG, Jung S, Lee YM, Lim J, Lee H, Lee J, Kim WJ. Programmed nanoparticle-loaded nanoparticles for deep-penetrating 3D cancer therapy. Adv Mater. 2018:e1707557. 10.1002/adma.201707557 29774603

[52] Priwitaningrum DL, Blondé JG, Sridhar A, van Baarlen J, Hennink WE, Storm G, Le Gac S, Prakash J (2016). Tumor stroma-containing 3D spheroid arrays: a tool to study nanoparticle penetration. J Control Release.

[53] Moore C, Jokerst JV (2019). Strategies for image-guided therapy, surgery, and drug delivery using photoacoustic imaging. Theranostics.

[54] Yu Z, Zhou P, Pan W, Li N, Tang B (2018). A biomimetic nanoreactor for synergistic chemiexcited photodynamic therapy and starvation therapy against tumor metastasis. Nat Commun.

[55] Yue C, Liu P, Zheng M, Zhao P, Wang Y, Ma Y, Cai L (2013). IR-780 dye loaded tumor targeting theranostic nanoparticles for NIR imaging and photothermal therapy. Biomaterials.

[56] Zhang C, Wang S, Xiao J, Tan X, Zhu Y, Su Y, Cheng T, Shi C (2010). Sentinel lymph node mapping by a near-infrared fluorescent heptamethine dye. Biomaterials.

[57] Wang S, Shang L, Li L, Yu Y, Chi C, Wang K, Zhang J, Shi R, Shen H, Waterhouse GIN (2016). Metal–organic-framework-derived mesoporous carbon nanospheres containing porphyrin-like metal centers for conformal phototherapy. Adv Mater.

[58] Ren H, Liu J, Su F, Ge S, Yuan A, Dai W, Wu J, Hu Y (2017). Relighting photosensitizers by synergistic integration of albumin and perfluorocarbon for enhanced photodynamic therapy. ACS Appl Mater Interfaces.

[59] Liu M, Zhang P, Deng L, Guo D, Tan M, Huang J, Luo Y, Cao Y, Wang Z (2019). IR780-based light-responsive nanocomplexes combining phase transition for enhancing multimodal imaging-guided photothermal therapy. Biomater Sci.

[60] Murphy MP, Smith RA (2007). Targeting antioxidants to mitochondria by conjugation to lipophilic cations. Annu Rev Pharmacol Toxicol.

[61] Chakrabortty S, Agrawalla BK, Stumper A, Vegi NM, Fischer S, Reichardt C, Kögler M, Dietzek B, Feuring-Buske M, Buske C (2017). Mitochondria targeted protein-ruthenium photosensitizer for efficient photodynamic applications. J Am Chem Soc.

[62] Zielonka J, Joseph J, Sikora A, Hardy M, Ouari O, Vasquez-Vivar J, Cheng G, Lopez M, Kalyanaraman B (2017). Mitochondria-targeted triphenylphosphonium-based compounds: syntheses, mechanisms of action, and therapeutic and diagnostic applications. Chem Rev.

[63] Zhang L, Yin T, Li B, Zheng R, Qiu C, Lam KS, Zhang Q, Shuai X (2018). Size-modulable nanoprobe for high-performance ultrasound imaging and drug delivery against cancer. ACS Nano.

[64] Li Y, Chen M, Yao B, Lu X, Song B, Vasilatos SN, Zhang X, Ren X, Yao C, Bian W (2020). Dual pH/ROS-responsive nanoplatform with deep tumor penetration and self-amplified drug release for enhancing tumor chemotherapeutic efficacy. Small.

[65] Xu F, Huang X, Wang Y, Zhou S (2020). A size-changeable collagenase-modified nanoscavenger for increasing penetration and retention of nanomedicine in deep tumor tissue. Adv Mater.

[66] Sakamoto K, Mikami H, Kimura J (2008). Involvement of organic anion transporting polypeptides in the toxicity of hydrophilic pravastatin and lipophilic fluvastatin in rat skeletal myofibres. Br J Pharmacol.

