# Supplementary material for: Mitochondria-targeted nanoplatforms for enhanced photodynamic therapy against hypoxia tumor
Source: J Nanobiotechnology. 2021 Dec 20;19:440. doi: 10.1186/s12951-021-01196-6 (PMC8686264; doi:10.1186/s12951-021-01196-6)
Supplement: Supplementary file 1 — Additional file 1: Fig. S1. Characterizations of 3BP@PLGA. Fig. S2. (a) UV–vis-NIR absorbance spectra of IR780 at elevated concentrations. (b) The relative absorbance of IR780 in the UV–vis spectrum at 789 nm. Fig. S3. (a) UV–vis-NIR absorbance spectra of 3BP at elevated concentrations. (b) The relative absorbance of 3BP in the UV–vis spectrum at 326 nm. Fig. S4. UV–vis-NIR absorbance spectra of 3BP@PLGA-IR780 and 3BP@PLGA. Fig. S5. Photo-stability study of 3BP@PLGA-IR780. Fig. S6. CLSM images of intracellular uptake of 3BP@PLGA-IR780 and 3BP@PLGA for MCF-7 and MBA-MD-231 cells. Fig. S7. (a) ATP standard curve and (b) ATP concentrations after different treatments. Fig. S8. Pharmacokinetics Study of 3BP@PLGA-IR780. Fig. S9. Ex vivo fluorescence imaging pictures of isolated tumors at 1, 3, 5, 7 and 10 days after intravenous injection of 3BP@PLGA-IR780. Fig. S10. PA signal changes of 3BP@PLGA-IR780 with PA laser excitation wavelengths ranging from 680 to 970 nm. Fig. S11. Representative tumor temperature change curve when receiving PDT (1.0 W cm−2, on 40 s, off to room temperature, 15 cycles). Fig. S12. Body-weight curves of six groups after various treatments. Fig. S13. H&E staining of the major organs of 4T1 tumor-bearing mice after different treatments. Fig. S14. (a) Hematological and blood biochemistry analysis of healthy mice after intravenous injection of 3BP@PLGA-IR780. (b) H&E staining of major organs of mice post injection of 3BP@PLGA-IR780. [file 12951_2021_1196_MOESM1_ESM.docx]

***Supplementary Information***

**Mitochondria-targeted Nanoplatforms for Enhanced Photodynamic Therapy against Hypoxia Tumor**

Jiexing Wen^1†^, Yong Luo^2†^, Hui Gao^1†^, Liang Zhang^3^, Xiang Wang^4^, Ju Huang^4^, Tingting Shang^3^, Di Zhou^5^, Dong Wang^6^, Zhigang Wang^3^, Pan Li^3^, Zhaoxia Wang^1^**^*^**


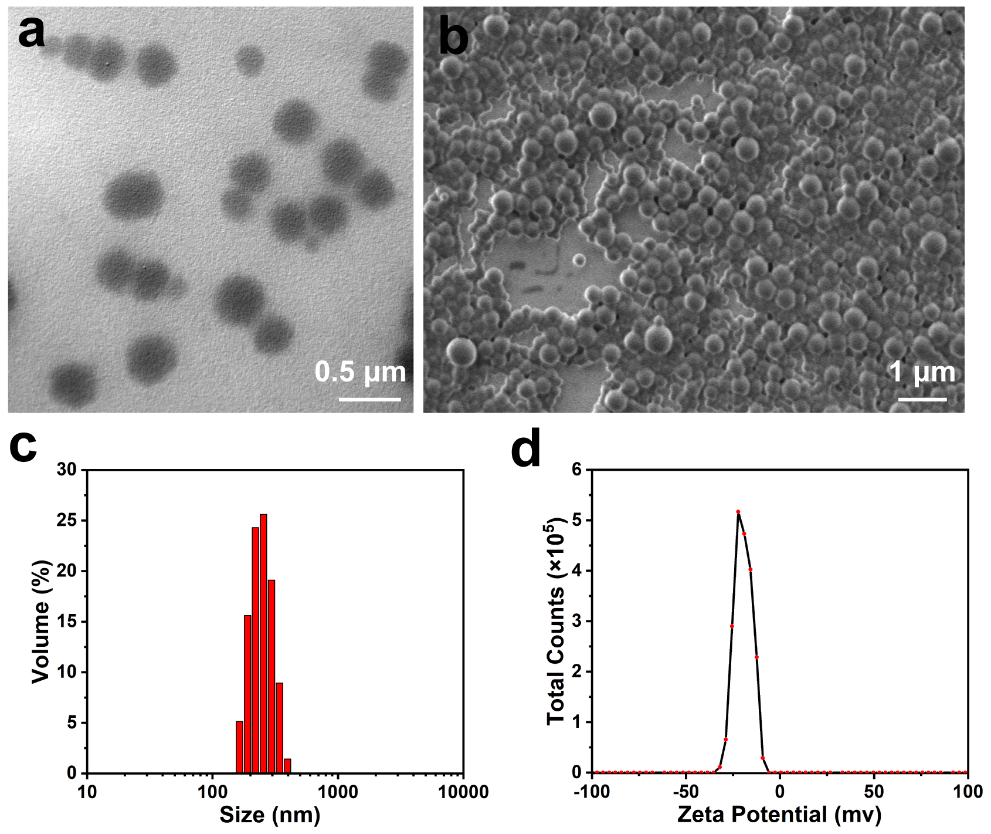


**Fig. S1**. Characterizations of 3BP@PLGA. (a) SEM image, (b) TEM image, (c) hydrodynamic diameter, (d) surface zeta potential of 3BP@PLGA.


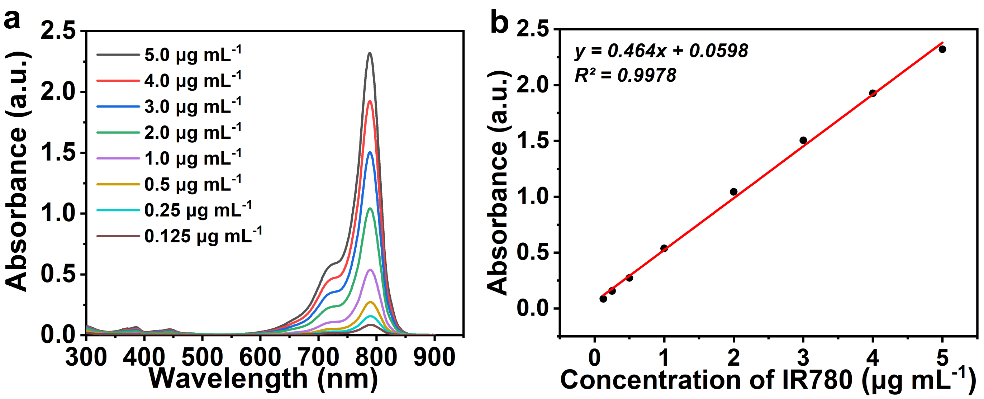


**Fig. S2** (a) UV-vis-NIR absorbance spectra of IR780 at elevated concentrations. (b) The relative absorbance of IR780 in the UV–vis spectrum at 789 nm.


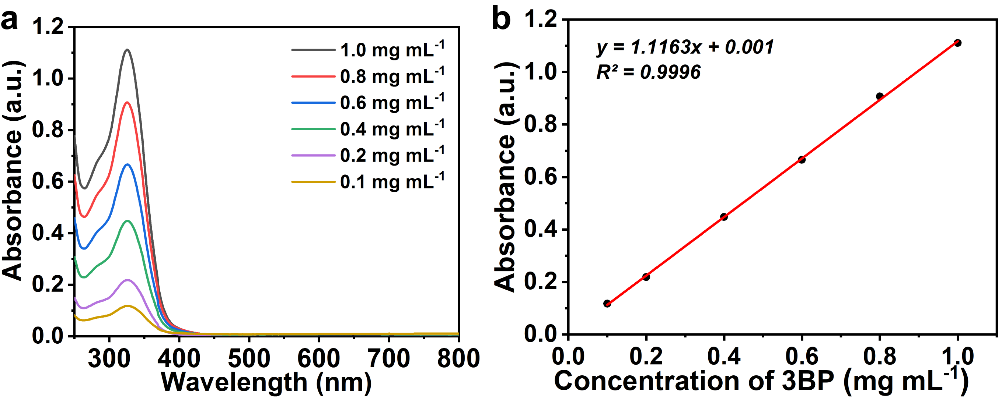


**Fig. S3** (a) UV-vis-NIR absorbance spectra of 3BP at elevated concentrations. (b) The relative absorbance of 3BP in the UV–vis spectrum at 326 nm.





**Fig. S4** UV-vis-NIR absorbance spectra of 3BP@PLGA-IR780 and 3BP@PLGA.


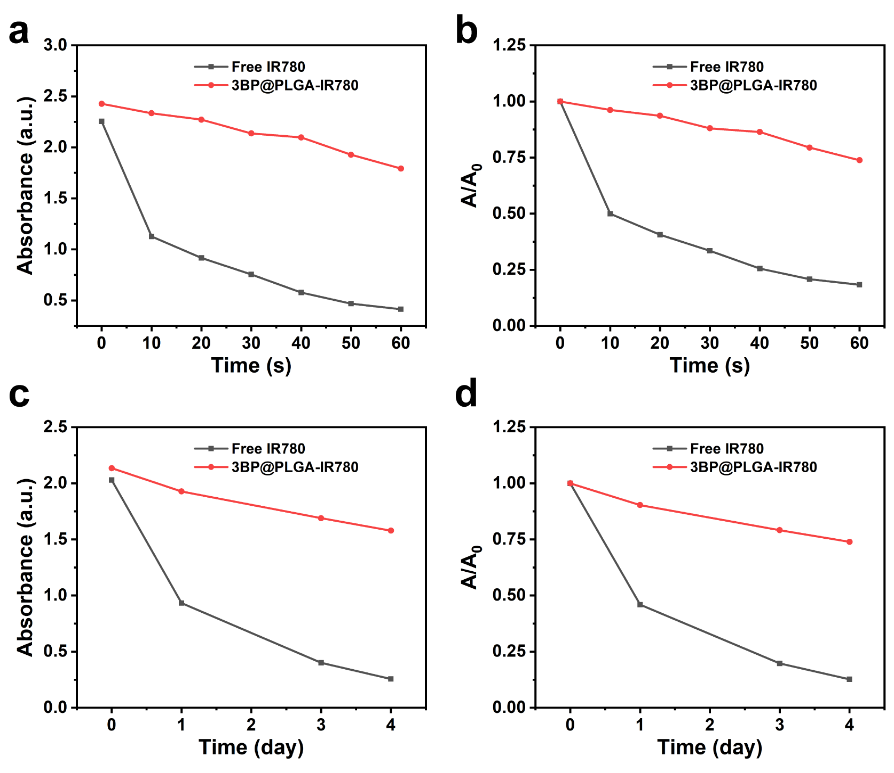


**Fig. S5** (a) Absorbance values of free IR780 and 3BP@PLGA-IR780 at 789 nm after laser irradiation (1.0 W/cm^2^). (b) The normalized absorption of (a). (c) Absorbance values of free IR780 and 3BP@PLGA-IR780 at 789 nm after various days stored in dark. (d) The normalized absorption of (c).


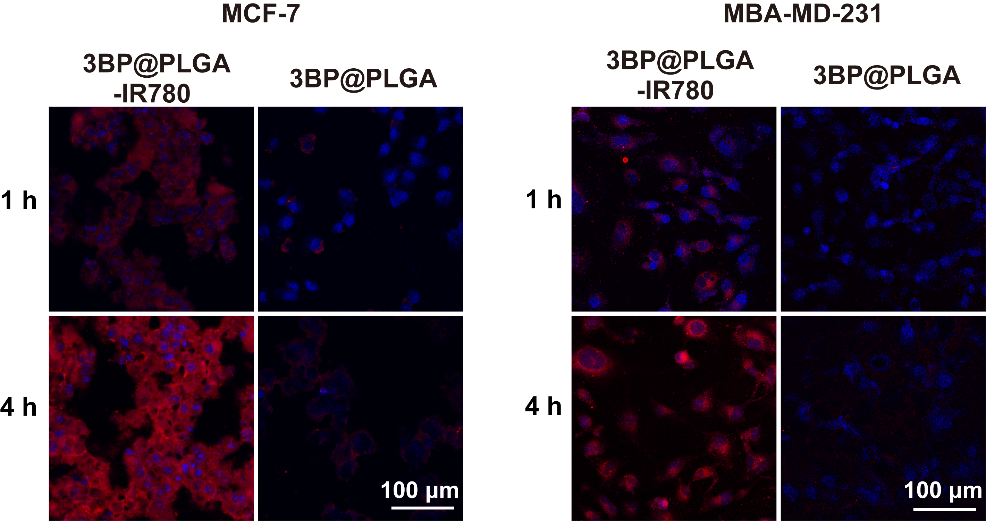


**Fig. S6.** CLSM images of intracellular uptake of 3BP@PLGA-IR780 and 3BP@PLGA for MCF-7 and MBA-MD-231 cells (the red represents nanoplatforms and the blue represents cell nucleus).


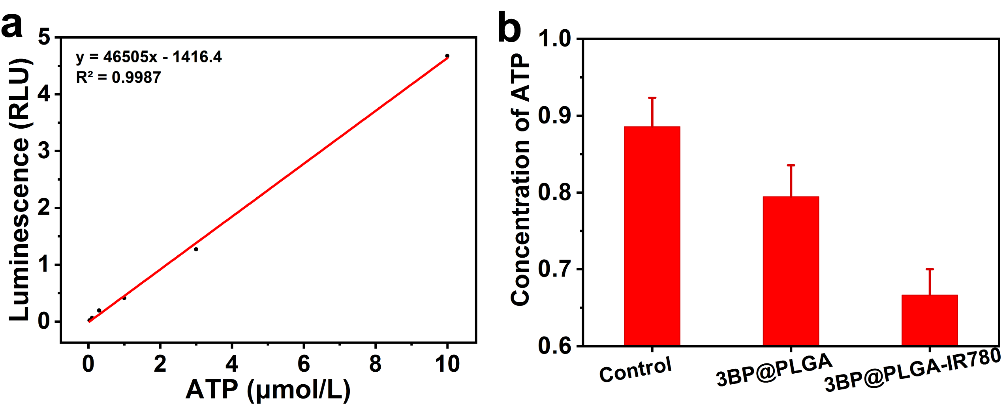


**Fig. S7** (a) ATP standard curve and (b) ATP concentrations after different treatments.


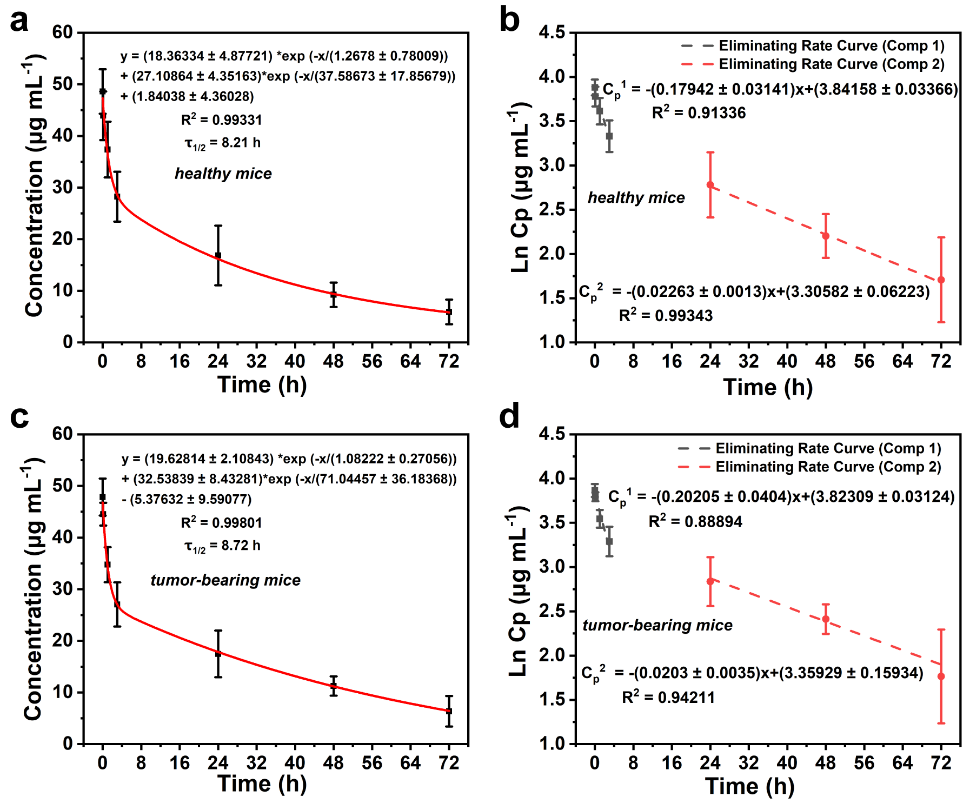


**Fig. S8** (a) IR780 concentrations in blood samples of healthy mice at different time points after intravenous injection of 3BP@PLGA-IR780 (n = 3) and (b) the corresponding time-course double-compartment elimination rate curve of 3BP@PLGA-IR780 in healthy mice. (c) IR780 concentrations in blood samples of 4T1 tumor-bearing mice at different time points after intravenous injection of 3BP@PLGA-IR780 (n = 3) and (d) the corresponding time-course double-compartment elimination rate curve of 3BP@PLGA-IR780 in 4T1 tumor-bearing mice.


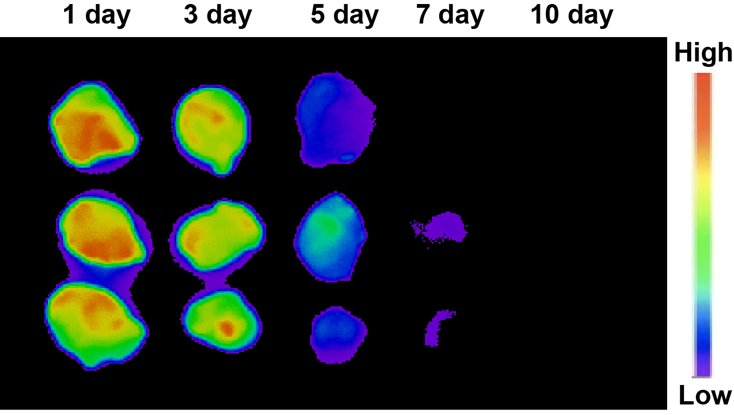


**Fig. S9** *Ex vivo* fluorescence imaging pictures of isolated tumors at 1, 3, 5, 7 and 10 days after intravenous injection of 3BP@PLGA-IR780.





**Fig. S10** PA signal changes of 3BP@PLGA-IR780 with PA laser excitation wavelengths ranging from 680 nm to 970 nm.


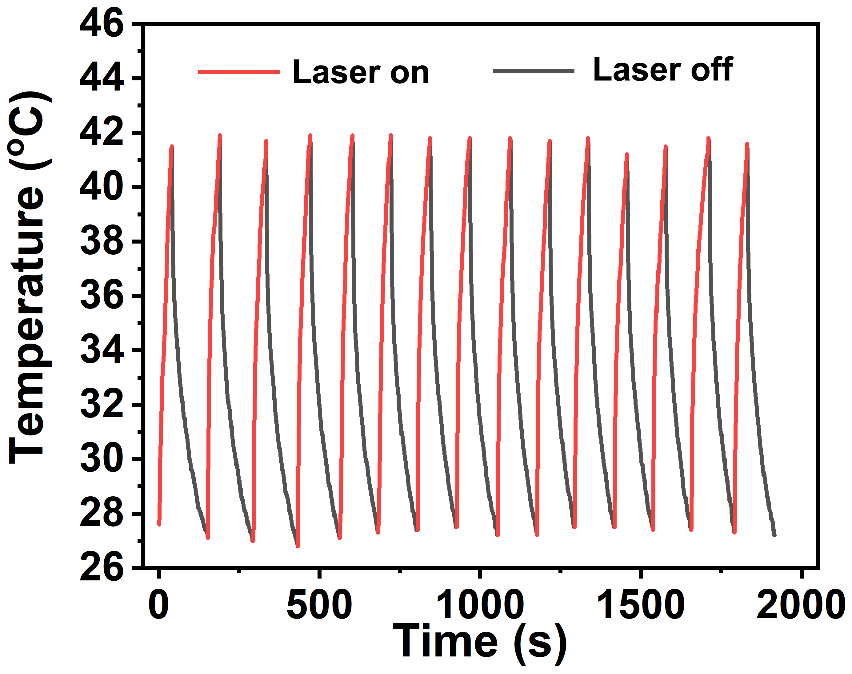


**Fig. S11** Representative tumor temperature change curve when receiving PDT (1.0 W cm^-2^, on 40 s, off to room temperature, 15 cycles).





**Fig. S12** Body-weight curves (n = 5) of six groups after various treatments.


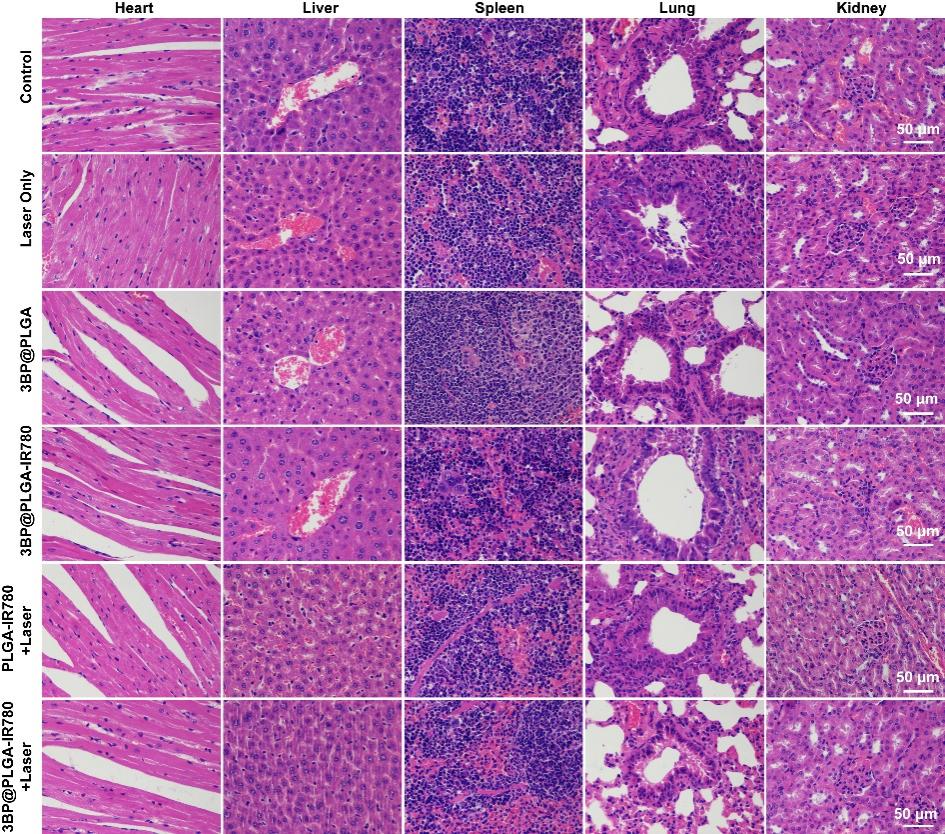


**Fig. S13** H&E staining of the major organs (heart, liver, spleen, lung and kidney) of 4T1 tumor-bearing mice after different treatments. The scale bars are 50 μm.


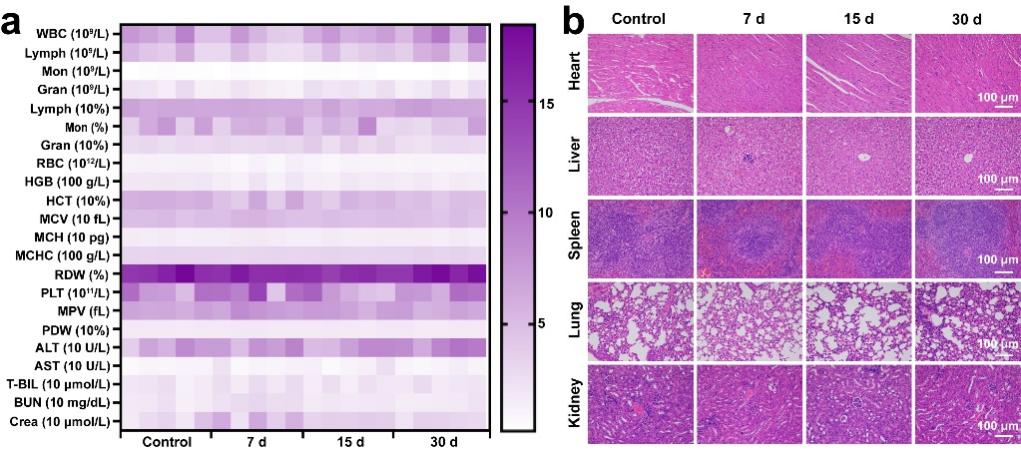


**Fig. S14.** (a) Hematological and blood biochemistry analysis of healthy mice after intravenous injection of 3BP@PLGA-IR780. (b) H&E staining of major organs (heart, liver, spleen, lung and kidney) of mice post injection of 3BP@PLGA-IR780.
